# Phenology and plasticity can prevent adaptive clines in thermal tolerance across temperate mountains: The importance of the elevation‐time axis

**DOI:** 10.1002/ece3.9349

**Published:** 2022-10-05

**Authors:** Luis Miguel Gutiérrez‐Pesquera, Miguel Tejedo, Agustín Camacho, Urtzi Enriquez‐Urzelai, Marco Katzenberger, Magdalena Choda, Pol Pintanel, Alfredo G. Nicieza

**Affiliations:** ^1^ Department of Evolutionary Ecology Estación Biológica de Doñana, CSIC Sevilla Spain; ^2^ Czech Academy of Sciences Institute of Vertebrate Biology Brno Czech Republic; ^3^ Laboratory of Bioinformatics and Evolutionary Biology, Department of Genetics Universidade Federal de Pernambuco Recife Prince Edward Island Brazil; ^4^ Department of Organisms and Systems Biology University of Oviedo Oviedo Spain; ^5^ Laboratorio de Ecofisiología and Museo de Zoología (QCAZ), Escuela de Ciencias Biológicas Pontificia Universidad Católica del Ecuador Quito Ecuador; ^6^ Biodiversity Research Institute (IMIB) University of Oviedo‐Principality of Asturias‐CSIC Mieres Spain

**Keywords:** local adaption, microclimate, niche conservatism, phenotypic plasticity, thermal tolerance, warming tolerance

## Abstract

Critical thermal limits (CT_max_ and CT_min_) decrease with elevation, with greater change in CT_min_, and the risk to suffer heat and cold stress increasing at the gradient ends. A central prediction is that populations will adapt to the prevailing climatic conditions. Yet, reliable support for such expectation is scant because of the complexity of integrating phenotypic, molecular divergence and organism exposure. We examined intraspecific variation of CT_max_ and CT_min_, neutral variation for 11 microsatellite loci, and micro‐ and macro‐temperatures in larvae from 11 populations of the Galician common frog (*Rana parvipalmata*) across an elevational gradient, to assess (1) the existence of local adaptation through a P_ST_‐F_ST_ comparison, (2) the acclimation scope in both thermal limits, and (3) the vulnerability to suffer acute heat and cold thermal stress, measured at both macro‐ and microclimatic scales. Our study revealed significant microgeographic variation in CT_max_ and CT_min_, and unexpected elevation gradients in pond temperatures. However, variation in CT_max_ and CT_min_ could not be attributed to selection because critical thermal limits were not correlated to elevation or temperatures. Differences in breeding phenology among populations resulted in exposure to higher and more variable temperatures at mid and high elevations. Accordingly, mid‐ and high‐elevation populations had higher CT_max_ and CT_min_ plasticities than lowland populations, but not more extreme CT_max_ and CT_min_. Thus, our results support the prediction that plasticity and phenological shifts may hinder local adaptation, promoting thermal niche conservatism. This may simply be a consequence of a coupled variation of reproductive timing with elevation (the “elevation‐time axis” for temperature variation). Mid and high mountain populations of *R. parvipalmata* are more vulnerable to heat and cool impacts than lowland populations during the aquatic phase. All of this contradicts some of the existing predictions on adaptive thermal clines and vulnerability to climate change in elevational gradients.

## Graphical abstract

This article is a representative and unique example of how adaptive thermal clines can be prevented by plasticity and phenological adjustments, with implications when evaluating thermal risk in temperate ectotherms to ongoing warming

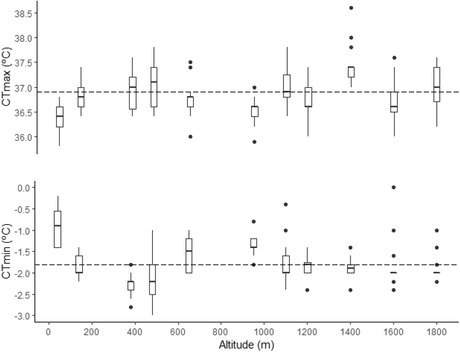

## 1 INTRODUCTION

Climate change is promoting fast increases in both mean temperatures and the frequency of extreme heat events and temporal anomalies, which may jeopardize biodiversity worldwide (IPCC, 2014; Pacifici et al., 2015; Parmesan, 2006). Species basically rely on four strategies to cope with this crisis: evolutionary changes in their tolerance limits, thermal acclimation (phenotypic plasticity), shifts in behavior and activity timing, and shifting geographical ranges in order to track historical climates (Habary et al., 2017; Hoffmann & Sgrò, 2011; Walther et al., 2002). Therefore, the study of population variation and phenotypic plasticity in physiological traits, and the correct characterization of thermal microenvironments can be decisive to predict the consequences of global warming (Camacho et al., 2015; Garland et al., 1991; Huey et al., 2012; Somero, 2010).

Spatial variation in thermal physiology (e.g., Critical Thermal Limits, CT_max_ and CT_min_) in relation to latitude and elevation has been thoroughly described at the interspecific level being often associated with environmental thermal heterogeneity (Bozinovic et al., 2011; Pintanel et al., 2019, 2022; Shah et al., 2017; Stevens, 1989; Sunday et al., 2019). Compared with longer range climatic variation of latitudinal gradients, elevational gradients change climate over shorter distances. That results in predictable changes in air temperatures between −6.5°C/km and − 3.5°C/km, due to adiabatic cooling, and an increase in thermal variability, associated with higher solar radiation and the lowering of air density (Hodkinson, 2005). These steeper climatic gradients may select for thermal adaptations to local conditions and thermal plasticity, and act as physiological barriers to gene flow potentially producing genetic differentiation, particularly in non‐seasonal tropical latitudes (Janzen, 1967; Polato et al., 2018). In turn, under moderate gene flow, populations living at divergent climates could introduce maladapted genotypes into each other, potentially decreasing the frequency of local adapted genotypes. This would determine a reduction in the steepness of the slope of adaptive clines, as it has been predicted theoretically (Endler, 1977; Slatkin, 1973), and demonstrated empirically in temperate amphibians (Bachmann et al., 2020). Recent intraspecific studies have revealed adaptive clinal variation with elevation in both CT_max_ and CT_min_ (Bishop et al., 2017; Klok & Chown, 2003; Sørensen et al., 2005) with more variation in CT_min_ than CT_max_ (Muñoz et al., 2014). In contrast, a number of studies did not reveal such elevational trend in physiological traits related to thermal tolerances (Buckley et al., 2013; Gvoždík & Castilla, 2001; Senior et al., 2019; Slatyer et al., 2016; Slatyer et al., 2019; Slatyer & Schoville, 2016; Tonione et al., 2020). Yet, reliable data to support adaptive clinal variation is still scant because of the need and difficulty of integrating phenotypic (P_ST_) and molecular divergence (F_ST_) (Brommer, 2011; Leinonen et al., 2008).

Several biotic and abiotic factors may prevent adaptive differentiation in thermal physiology when analyzing mountain clines. Most animals experience climate at fine‐scale patches, and microenvironmental temperatures actually faced by the organism can deviate greatly from recorded mean air temperatures obtained at larger spatial scales (Helmuth, 2009; Potter et al., 2013; Suggitt et al., 2011). In fact, recent analyses suggest that macroclimatic variables (e.g., WorldClim, Hijmans et al., 2005) may only weakly predict tolerance limits and physiological niches compared with microclimatic variables (Farallo et al., 2020; Gutiérrez‐Pesquera et al., 2016; Katzenberger et al., 2018; Navas et al., 2013; Pintanel et al., 2019, 2022).

Spatiotemporal changes in microclimate may result, for example, from differences in topography and vegetation cover (Porter et al., 2002). Since microclimatic heterogeneity cannot be captured by downscaling regional climatic variation (Caillon et al., 2014; Diamond & Chick, 2018; Navas et al., 2013; Pincebourde et al., 2016), it becomes important to measure it. This is particularly important for assessing climatic tolerance in animals living at buffered microhabitats, like ponds (Ex. tadpoles, Gutiérrez‐Pesquera et al., 2016; Katzenberger et al., 2018; Pintanel et al., 2022). This thermal heterogeneity at the microclimatic scales allows organisms for behavioral thermoregulation, which may preclude the evolution of physiological adaptations in performance by reducing the exposure of organisms to extreme temperatures (i.e., the Bogert effect; Huey et al., 2003, 2012; Kearney et al., 2009; Buckley et al., 2015; Farallo et al., 2018; Muñoz, 2022). Otherwise, organisms may be non‐active year‐round, adopting dormant physiological states such as diapause, hibernation or estivation, in order to escape stressful extreme temperatures (Ragland & Kingsolver, 2008), or because breeding habitats or resources are temporarily unavailable (e.g., ice covered aquatic habitats, and pond drying). Temporal adjustments of activity can be the result of two additive components, one seasonal (or phenological) and one at a finer temporal scale (24‐h) that can be dependent on season and particular weather conditions. The limit for these adjustments will be imposed by the energy demand (Kearney & Porter, 2017, 2020).

Therefore, in absence of energetic constraints, populations can persist at their spatial locations despite change in thermal conditions, without changes in physiological traits. Thus, phenological adjustments in activity can modify the strength of directional selection over thermal tolerance limits through altitudinal gradients (Álvarez et al., 2012; Phillimore et al., 2010; Socolar et al., 2017). This is important because the physiological adjustment of thermal traits may be subjected to severe constraints. For instance, extreme heat stress can occur even at high elevations (Sunday et al., 2014), so populations living in mountain areas should face both heat and cold extremes, which leads to an unlikely solution (“master‐of‐all” superorganism, Remold, 2012).

All these factors can contribute to buffer the realized thermal variation (i.e., the range of effective temperatures experienced by individuals) along elevational gradients and, ultimately, they can promote the pervasiveness and retention of climatic niches; hence, organisms would maintain their thermal niches unchanged while moving along an elevation gradient. In this context, behavioral tracking of the microclimatic niche over space and phenology can allow for retention of the microclimatic niche rather than adapting to the new local conditions with changes in physiology (Farallo et al., 2020; Huey et al., 2003; Kearney et al., 2009; Muñoz, 2022).

Populations living in highly variable thermal environments would express greater plasticity in their thermal tolerances (Angilletta, 2009; Gunderson & Stillman, 2015; Chevin & Hoffmann, 2017; Mallard et al., 2020; but see, for deeper discussion, Gilchrist, 1995, Enriquez‐Urzelai et al., 2020). Besides behavioral thermoregulation and phenological adjustments, thermal acclimation may also prevent directional selection on physiological thermal traits, which would constrain thermal adaptation to the new climatic conditions. In fact, organisms can retain plasticity enough to allow for rapid shifts in thermal tolerances, thus precluding thermal stress, death and, likely, the effects of natural selection (Chevin et al., 2010; Huey et al., 2012; Levins, 1969).

**FIGURE 1:**
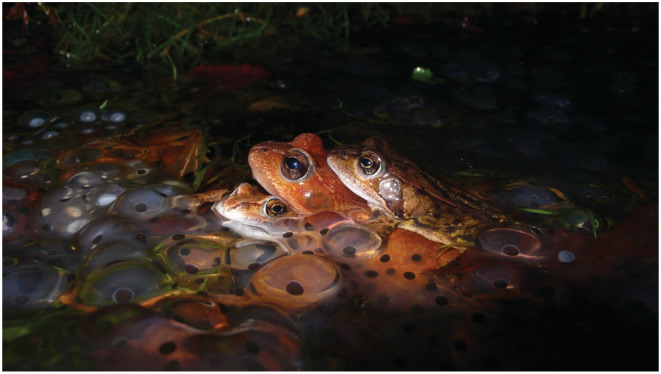
Amplectant pair of *Rana parvipalmata* and a satellite male surrounded by masses of recently spawned eggs and embryos in a breeding nucleus in the Color Valley (380 m a.s.l.). A second amplexus is under the egg masses. Breeding occurs in a series of small, shallow spring pools and ditches (maximum depth <12 cm) under complete canopy cover.

Here we examined elevational clinal variation in the upper (CT_max_) and lower (CT_min_) critical thermal limits and their plasticity in 11 populations of the Galician common frog, *Rana parvipalmata* (Figure 1), across an altitudinal gradient from 40 to 1800 m a.s.l. We focus on the aquatic tadpole stage because the breeding aquatic habitat of many amphibian species exhibit low thermal heterogeneity. This limits tadpole ability for behavioral thermoregulation compared with terrestrial adult stages (see Feder & Hofmann, 1999) and, thus, determining that thermal selection on the aquatic phase could be a major driver of tadpole variation in critical thermal limits and its plasticity. In addition, recent research has identified maximum pond temperature as an important range‐limiting factor for *R. temporaria* / *Rana parvipalmata* (Enriquez‐Urzelai, Kearney, et al., 2019). Second, we analyzed population vulnerability to thermal stress by estimating warming and cooling tolerances (sensu Sunday et al., 2014; Gutiérrez‐Pesquera et al., 2016). Since average temperatures usually decline with elevation, and thermal physiology limits are driven by peak environmental temperatures (Buckley & Huey, 2016; Gutiérrez‐Pesquera et al., 2016; Overgaard et al., 2014; Pintanel et al., 2019), we posit the following two predictions: (1) greater heat impacts are expected at the lowlands (Pintanel et al., 2019; Sunday et al., 2014), whereas higher cold impacts are forecasted for mountain populations (Pintanel et al., 2019; Sunday et al., 2014) (*elevational thermal vulnerability hypothesis*); and, therefore, (2) under a scenario of selection in CTs, higher elevation populations will evolve lower tolerances to high temperatures and higher tolerances to low temperatures *(elevational thermal adaptive hypothesis*). In addition, we analyzed the among‐populations variation in acclimation capacity (i.e., as a form of phenotypic plasticity) for thermal tolerances and the potential for local adaptation in thermal limits (CT_max_ and CT_min_) by means of P_ST_‐F_ST_ comparisons across these populations. Considering that populations at different elevations may be exposed to different extreme temperatures and variable thermal ranges, we hypothesize that these populations will also differ in acclimation ability.

## 2 MATERIALS AND METHODS

### 2.1 Study system, population breeding dynamics, and sampling

The Galician common frog (*Rana parvipalmata*), formerly part of the European common frog (*Rana temporaria*) complex (Dufresnes et al., 2020), is endemic to the northwest Iberian Peninsula. Based on projected rates of climate change, there is growing evidence that common frogs (*Rana temporaria* and *Rana parvipalmata*) in northwestern Iberia may experience heat stress associated with heat waves in coming decades (Enriquez‐Urzelai et al., 2020; Enriquez‐Urzelai, Sacco, et al., 2019), although the impact on population growth would depend on the potential for behavioral thermoregulation of adults and, especially, juvenile individuals (Enriquez‐Urzelai et al., 2018, 2020; Enriquez‐Urzelai, Sacco, et al., 2019). Anyhow, recent forecasts based on climatic niche models (both correlative and mechanistics approaches), predicted alarming decreases in climatic suitability in genetic “hotspots” of the *R. temporaria* / *R. parvipalmata* complex (e.g., northern Iberian Peninsula and other glacial refuges in southern Europe), and, under the worst scenarios, the extinction of *R*. *parvipalmata* and the Cantabrian *R*. *temporaria* along with many populations in central Europe (Enriquez‐Urzelai, Kearney, et al., 2019). In fact, under the most extreme climate change scenario, all mountain ranges but the Alps will also become thermally unsuitable by 2070 (Enriquez‐Urzelai, Kearney, et al., 2019).

**FIGURE 2:**
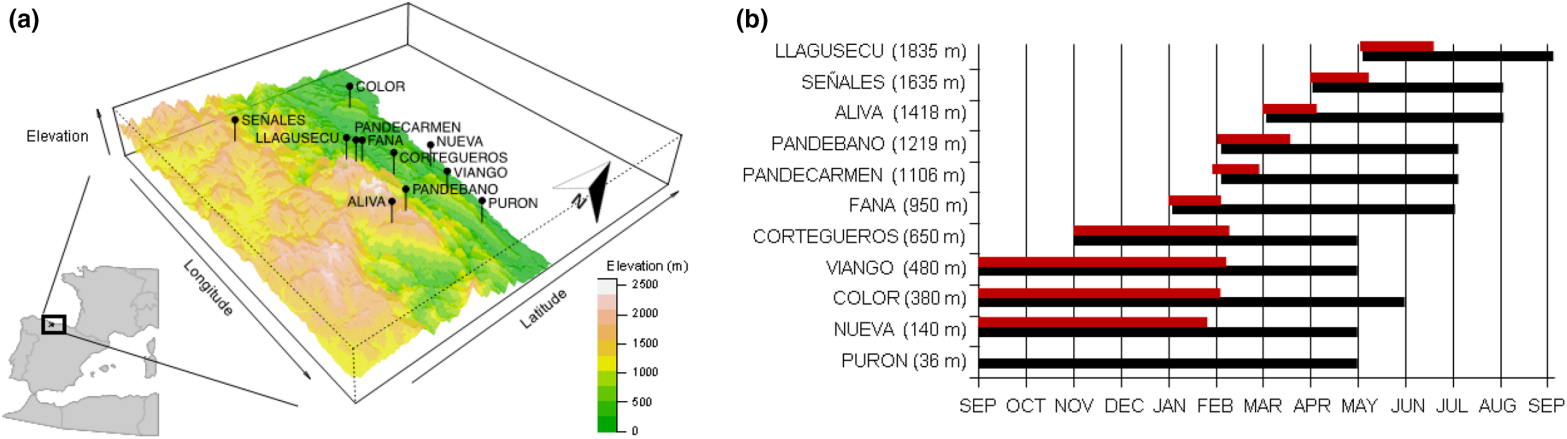
(a). Study area and geographic locations of 11 populations of *Rana parvipalmata* (sample points for embryos). (b) Observed temporal variation in the breeding and larval period of *Rana parvipalmata* along the altitudinal gradient for the analyzed populations. Bars marked in red indicates population adult breeding activity; black bars show the presence of larvae in the water. Purón adult breeding activity is not marked because the scarcity of observations.

Reproductive timing is strongly conditioned by elevation which results in a wide, sequential breeding period over most of the year (Figure 2; Álvarez et al., 2012). The observed breeding phenology seems to be the result of physical constrains (e.g., pond desiccation, winter severity at high elevation) derived from climatic conditions, and selection on adults to prevent breeding out of time that renders reproductive success, rather than the product of natural selection acting on larval thermal physiology. In the lowlands, breeding take place in autumn, extends over weeks or a few months with spawning peaks associated with heavy rains, and metamorphs leave the aquatic habitat by late‐winter and early spring, before the risk of pond desiccation increases. In contrast, above 1400 m a.s.l. the onset of reproduction delays until snow melting in spring, populations show explosive breeding (lasting 1–2 weeks), and the larval phase can extend until the end of summer (Figure 2). For mountain top populations (1800–2200 m a.s.l.), the seasonal activity window may be less than 5 months to carry out larval development, juvenile growth, and fat storage before the onset of hibernation. Sampling was carried out between 2012 and 2014 in Picos de Europa National Park and surrounding areas, covering a relatively reduced geographical area (Figure 2, Table S2). We selected a total of 11 populations along an elevational transect between 40 and 1800 m a.s.l. All of them belong to the T2 (eastern) lineage of *Rana parvipalmata* (Dufresnes et al., 2020) and were assigned to a maximum of five genetic clusters (Choda, 2014).

For each site, we haphazardly collected 5–7 recently fertilized clutches of *R. parvipalmata* to obtain a representative sample of the population. Embryos were transported to the Doñana Biological Station (EBD‐CSIC), and maintained inside climatic chambers (FitoClima, Aralab) under constant conditions of photoperiod (12:12 L:D) and temperature (15°C) until hatching. Thereafter, tadpoles were kept in plastic containers at a larval density of 20 individuals · L^−1^ and constant photoperiod (12:12 L:D) and temperature (15°C), until they reached stage 26 (Gosner, 1960) and the tests were conducted. Since we assumed no cross‐generational effects, this common garden approach allowed us to disentangle the effects of environment and genetics on physiological thermotolerance.

Animal captures were carried out under permits granted by Government of the Principality of Asturias (2010/000371), and Picos de Europa National Park (CO/09/121/2012, CO/09/0125/2013, CO/09/012/2014). All procedures complied with the country legal requirements on animal welfare (RD 53/2013) and were conducted in accordance with the guidelines of the Research Ethics Committee of the University of Oviedo under authorization #8‐INV‐2012. The members of the research team have approved licenses by the Service of Animal Welfare and Production of the Principality of Asturias to design and conduct experimental protocols with animals (license types C and D to A.G.N). This study was carried out in compliance with the ARRIVE guidelines (Animal Research: Reporting in Vivo Experiments) for how to report animal research in scientific publications (https://arriveguidelines.org/arrive‐guidelines).

### 2.2 Environmental data

To better characterize potential selective pressures that might lead to thermal local adaptation, we obtained micro‐ and macro‐environmental thermal data for all the sampled populations. To define the thermal profile at each site, we took into account the population's phenology, encompassing both the breeding and larval periods (Figure 2; Tables S1–S5). In order to characterize the macroclimatic thermal environments, we used the ‘extract’ function in the R package raster (Hijmans & van Etten, 2014; R Core Team, 2019) to obtain temperature data from WorldClim 2 layers (the temperature of shaded air at around 2 m off the ground, 30 s or 1 km^2^ spatial resolutions; records from 1970 to 2000) (Fick & Hijmans, 2017). For each population, we assessed monthly maximum (TMAX) and minimum (TMIN) temperatures restricted to the time period with presence of larvae in the ponds (Tables S2 and S3). In addition, we calculated seasonal temperature range (SR) as the difference between TMAX and TMIN (Supporting Information, Table S4). Since most animals experience climate at fine‐scale patches (Porter et al., 2002; Suggitt et al., 2011), macroclimatic data may be poorer predictors of thermal physiology than microclimatic variables (Gutiérrez‐Pesquera et al., 2016; Katzenberger et al., 2018; Pintanel et al., 2019; Sunday et al., 2014). Hence, we gathered microclimatic data by placing HOBO Pendant temperature dataloggers that recorded temperature every 10–30 min at each pond (Table S5). All the Hobo data‐loggers were placed underwater on the bottom of the ponds at a depth of 15–25 cm. One datalogger (Aliva population, 1420 m) was lost and we employed the information from a nearby population with similar elevation and pond characteristics (Pandébano, 1220 m). For each location, we calculated maximum daily temperature (tmax), minimum daily temperature (tmin), seasonal thermal range (sr) (sr = tmax‐tmin) and average daily temperature range (dr) for the period when tadpoles are present in the ponds (Figure 1, Table 1, Table S5).

**TABLE 1:** Critical thermal limits (CT_max_ and CT_min_, *N*, Mean and Standard Error), estimated at a pre‐assays acclimation temperature of 20°C. Maximum (TMAX, tmax) and minimum (TMIN, tmin) environmental temperatures (in caps, WorldClim estimator, in lowercase microclimate estimators). Average seasonal (SR: TMAX‐TMIN; sr: tmax‐tmin) and diel (dr: daily tmax‐tmin) temperature ranges.

Population	Elevation (m)	CT_max_ (°C)			CT_min_ (°C)			TMAX	tmax	TMIN	tmin	SR	sr	dr	WT	wt	CT	ct
		N	$\bar{X}$	SE	N	$\bar{X}$	SE											
Llagusecu	1835	14	37.0	0.1	14	−1.9	0.1	14.1	29.7	1.4	0.5	5.4	29.2	5.5	22.9	7.3	3.3	2.4
Señales	1635	14	36.8	0.1	16	−1.8	0.1	15.6	22.6	−1.2	4	7.0	18.6	4.9	21.2	14.2	0.6	5.8
Aliva	1418	14	37.5	0.1	16	−1.9	0.1	16.3	24.5	−0.5	4.3	6.3	20.2	6.5	21.2	13.0	1.4	6.2
Pandébano	1219	16	36.7	0.1	16	−1.9	0.1	15.4	28.9	−0.8	0.6	6.9	28.3	7.8	21.3	7.8	1.1	2.5
Pandecarmen	1106	16	37.0	0.1	16	−1.8	0.1	16.8	33	−0.3	0.2	7.5	34.6	13.1	20.2	4.0	1.5	2.0
Fana	950	16	36.5	0.1	16	−1.3	0.1	17.2	29.3	−0.2	1.5	7.4	27.8	8.9	19.3	7.2	1.1	2.8
Cortegueros	650	16	36.8	0.1	12	−1.6	0.1	18.9	28.4	1.8	1	7.5	27.4	6.2	17.9	8.4	3.3	2.6
Viango	480	16	37.1	0.1	15	−2.1	0.2	19.3	25.6	1.9	2.4	7.0	23.2	5.0	17.8	11.5	4	4.5
Color	380	16	36.9	0.1	16	−2.3	0.1	19.1	22.5	3	3.8	6.7	18.7	1.0	17.8	14.4	5.3	6.1
Nueva	140	16	36.8	0.1	13	−1.9	0.1	21.1	15.7	5	6.4	7.1	9.3	1.7	15.7	21.1	6.9	8.3
Purón	36	16	36.4	0.1	16	−0.9	0.1	21.7	11.4	4.7	9.9	7.6	1.5	0.4	14.7	25.0	5.6	10.8

Warming tolerance (WT, wt; WT = CT_max_‐TMAX, wt = CT_max_‐tmax,) and cooling tolerance (CT, ct; CT = TMIN‐CT_min_, ct = tmin‐CT_min_) for eleven populations of *R. parvipalmata*. Data on geographical locations appear in Tables S1–S3. All environmental data were obtained only during the breeding period of each population

### 2.3 Estimation of critical thermal limits and warming and cooling tolerances

Previous research with tadpoles showed that there is not significant variation in CTs across the larval period and only later on, at the verge of metamorphosis climax (Gosner stage 42, Gosner, 1960), both CTs exhibit a strong decline (e.g., Floyd, 1983). Therefore, to determine the CT_max_ and CT_min_ of *R. parvipalmata* populations, we haphazardly selected 32 larvae within Gosner stages 26–39 from each population pool (a mix of 5–7 families per population). Then, each population sample was split into two groups of 16 tadpoles for the estimation of CT_max_ and CT_min_, respectively. Tadpoles were kept individually in 400 ml plastic cups and acclimated inside environmental chambers (FitoClima, Aralab) to a constant temperature of 20°C with a photoperiod of 12 L:12D for 4 days before conducting the tolerance assays. This is the time required for adult amphibians and in tadpoles (J. Turriago and M. Tejedo, unpublished data) to stabilize CT_max_ after a large change in environmental temperature, such as field and laboratory conditions (Hutchison, 1961). Tadpoles were fed ad libitum with Purina rabbit chow. Oxygen saturation in the vessels was daily monitored with a laboratory multi‐parameter probe (WTW CellOx® 325) and recorded values were always over 60%. Thermal tolerance limits (CT_max_ and CT_min_) were determined using the Hutchison's dynamic method (Gutiérrez‐Pesquera et al., 2016; Lutterschmidt & Hutchison, 1997b). Tadpoles were weighed immediately before the beginning of the test and placed in individual 100 ml containers with dechlorinated tap water inside a thermal bath of 15 L (HUBER K15‐cc‐NR) previously stabilized to 20°C (acclimation temperature and start temperature) for five minutes. Afterwards, each animal was exposed to a constant heating / cooling rate (ΔT = 0.25°C min^−1^; CT_max_ and CT_min_, respectively) until the endpoint was attained. The endpoint for both thermal limits was defined as the temperature at which tadpoles become motionless and failed to respond to external stimuli (10 consecutive gentle prods with a wooden stick applied in 2 s intervals). Since tadpoles were small in size, we assumed that body temperature was equivalent to water temperature (Gutiérrez‐Pesquera et al., 2016; Lutterschmidt & Hutchison, 1997a). Hence, CT_max_ and CT_min_ were recorded as the water temperature beside the tadpole, using a Miller & Weber quick‐recording thermometer (to the nearest 0.1°C). After the tolerance limit was determined, we immediately transferred tadpoles to water at 20°C, to allow for recovery. Each tadpole was tested only once, and its survival was assessed a few minutes and 24 hours after the experimental procedure. Only those individuals who remained alive and exhibited normal behavior 24 h after the test were included in subsequent analyses.

For each population, warming tolerances (Deutsch et al., 2008; Duarte et al., 2012) were defined as the difference between CT_max_ and the maximum exposure environmental temperature (mean maximum temperature for the warmest month) taken at either the micro‐ (pond; wt = CT_max_–tmax) or the macro‐scale (air; WT = CT_max_‐TMAX). By doing so, we expect to reduce noise associated with extremely low or high temperatures during periods when larval habitat is lacking (dry or frozen ponds) and thus obtain more plausible estimates of thermal hazard (see also Duarte et al., 2012; Gutiérrez‐Pesquera et al., 2016; Hoffmann et al., 2013). Similarly, cooling tolerances (ct, CT) were determined as the difference between the minimum environmental temperature taken at either the micro scale pond temperature (tmin), or the macro scale minimum air temperature (TMIN), and population CT_min_ (tmin–CT_min_ and TMIN‐ CT_min_, respectively) (Gutiérrez‐Pesquera et al., 2016; Pintanel et al., 2019; Slatyer et al., 2016; Sunday et al., 2014). We used both WorldClim air temperature and microenvironmental water temperatures, recorded only during the larval period of each population.

### 2.4 Phenotypic plasticity in CT_max_ and CT_min_

We studied temperature acclimation in both thermal limits (CT_max_ and CT_min_) of five populations, corresponding to low: Nueva (140 m), Cortegueros (650 m); medium: Pandecarmen (1106 m), Aliva (1418 m), and high: Llagusecu (1835 m) elevations. For each population, tadpoles were individually maintained in 400 ml plastic vessels and randomly separated into four batches, totaling 32 tadpoles per batch. Each batch was then acclimated to a specific constant temperature (6, 13, 20 or 27°C) with a 12 L:12D photoperiod, for four days (Table S6). These temperatures are relevant because they cover the thermal range of tadpole exposure along the elevation gradient (Álvarez et al., 2012). Although this range can be exceeded in natural conditions at some point, it represents a reasonable adjustment for acclimation. We set the target temperature treatments in a Binder thermal chamber for 6°C treatments, and FitoClima chambers to obtain the rest of thermal treatments. Additionally, we employed Portable Fluid Heaters with Regulation Adjustment, (patent licensing U201431698) to reduce variability in water temperatures within thermal treatments (see caption in Figure 6). CT_max_ and CT_min_ were determined following the above protocols with a start temperature of 20°C for all acclimation temperatures.

### 2.5 Molecular markers. DNA extraction

In order to examine the potential for local adaptation in thermal limits (CT_max_ and CT_min_), we conducted P_ST_‐F_ST_ comparisons across the 11 populations by using 11 microsatellite loci to assess neutral variation (Table S7). We chose microsatellites because these markers are suitable to define population structure at the fine scale (Camacho‐Sánchez et al., 2020; DeFaveri et al., 2013; Lemopoulos et al., 2019; Saint‐Pé et al., 2019). To reduce the likelihood of including closely related individuals, we obtained the material for genetic analyses from breeding adults, either as buccal swabs (Pidancier et al., 2003) or by cutting the tip of a toe on the foot. In the few cases where tadpoles or embryos were sampled, each tadpole was collected at a different pool to avoid sampling of highly related individuals. All samples were stored at low temperature in 99% EtOH. Whole genomic DNA was isolated from samples with either standard Chelex extraction (500 μl of a 10% Chelex solution [Chelex‐100, Bio‐Rad] incubate with 7 μg Proteinase K at 55°C for 60 min and 100°C for 20 min) or an E.Z.N.A kit for DNA extraction. We selected 11 polymorphic microsatellite loci whose primers were developed for *Rana temporaria*. These markers included different degrees of polymorphism (Supporting Information S7).

### 2.6 Estimates of neutral genetic and phenotypic divergences (F_ST_ and P_ST_ )

We used the MICRO‐CHECKER software (Oosterhout et al., 2004) to check for genotyping errors and null alleles. No evidence of scoring alleles and large alleles dropout was found, but RtU7 was excluded due to the possibility of null alleles. In addition, four markers (Rtempμ1, RtU4, RtμH, and RtμB) were discarded due to failed amplifications. The remainder six markers (Rtempμ2, Rtempμ4, BFG072, BFG093, BFG183, and BFG241) were quantifiable for the 11 experimental populations and therefore used to estimate F_ST_ values. Exact tests for departure from Hardy–Weinberg equilibrium (HWE) and tests for linkage disequilibrium were performed for each population across all loci, and at each locus individually, using GENEPOP v2.1 (Raymond & Rousset, 1995). Significance was evaluated using the Markov chain methods (Guo & Thompson, 1992) with 5000 dememorizations steps and 1000 batches of 10,000 interactions per batch for HWE, and 5000 interactions for linkage disequilibrium tests.

To test whether local adaptation or neutral divergence drive the elevational variation in CT_max_ and CT_min_, we compared the genetic differentiation (F_ST_) and the genetic divergence of quantitative traits (Q_ST_) among populations, which allow to discern whether trait differentiation is due to genetic drift or natural selection (Leinonen et al., 2008). F_ST_ estimates were calculated according to Weir and Cockerham (1984), using FSTAT 2.9.3.2 (Goudet, 2002). As a surrogate for Q_ST_ data, we used a measure of phenotypic divergence of a trait (P_ST_) (Brommer, 2011), which can be calculated by the equation:

$$P_{\mathrm{ST}}=\frac{c\sigma_{B}^{2}}{c\sigma_{B}^{2}+2h^{2}\sigma_{w}^{2}}$$

where $\sigma_{B}^{2}$ is the phenotypic variance between populations, $\sigma_{w}^{2}$, the phenotypic variance within‐ population, and $h^{2}$ is the character heritability. The constant *c* represents the proportion of the total variance due to additive genetic effects across populations (Leinonen et al., 2006).

To obtain the P_ST_ values for each pair of populations, we used a linear mixed‐effects model (LMM), with population defined as a random factor, using the lme4 r‐package: CT_max_ ~ 1 + (1|Population), and CT_min_ ~ 1 + (1|Population) (Bates et al., 2015). We used the error variance as a proxy for $\sigma_{w}^{2}$ (within‐population variance) and the intercept variation for $\sigma_{B}^{2}$ (variance between populations). Thus, P_ST_ estimates depend on the ratio $\frac{c}{h^{2}}$. Since these parameters are extremely challenging to obtain in the wild and usually unknown (Pujol et al., 2008), we considered a set of values to calculate P_ST_ (Brommer, 2011). We constructed several matrices for the P_ST_ values obtained for different values of *c* and $h^{2}$. For each possible combination, the overall mean values (overall P_ST_) and their 95% confidence intervals were calculated using a nonparametric bootstrap and compared with the upper limit of the confidence interval for overall F_ST_ (Supporting Information S7–S9). The value of the *c/h* ^2^ ratio at which the lower confidence interval for P_ST_ and the upper F_ST_ estimates overlap, can be interpreted as a measure of the robustness of the difference between F_ST_ and P_ST_ estimates (see Brommer, 2011). The use of P_ST_ presents several caveats, since non‐additive genetic variance (epistasis or dominance effects), maternal effects or environmental factors and genotype‐environment interaction, can lead to a distorted picture of additive genetic variation when studying only phenotypic variation in natural conditions (Brommer, 2011; Leinonen et al., 2008; Pujol et al., 2008). However, because experimental individuals were raised and analyzed under the same environmental conditions, we can assume a lower risk of unwanted effects.

### 2.7 Statistical analyses

To test for geographic variation in CT_max_ and CT_min_ among populations of *R. parvipalmata*, we fitted two separate ANCOVA models for CT_max_ and CT_min_, as dependent variables, including populations as categorical factor, and body mass as a covariate. Tukey HSD post‐hoc tests were conducted to identify which populations differed in their thermal limits.

To test for covariation between CT_max_ and CT_min_, thermal variation and elevation, we assessed linear and quadratic regression models using elevation and the thermal data as independent variables and CT_max_, CT_min_, WT, wt, CT, ct as dependent variables. This allowed to determine the relationship between species' physiological limits and environmental thermal predictors, including elevation. Paired t‐Test and non‐parametric test of Kolmogorov–Smirnov (KS) were used to assess differences between estimates of vulnerability of exposure to extreme temperatures, determined using macroclimate (WT, CT) or microclimate (wt, ct) thermal data. Finally, we ran Mantel tests with 999 permutations in the R‐package ‘vegan’ (Oksanen et al., 2018) to test the correlations between P_ST_ and F_ST_ pairwise population matrices.

In order to evaluate whether acclimation effects differ among populations, we used a two‐way analysis of variance of CT_max_ and CT_min_, with temperature (6, 13, 20, and 27°C) and population as fixed factors. We estimated the Acclimation Response Ratio (ARR) that measures the change in thermal tolerance relative to change in acclimation temperature (Claussen, 1977; Ruthsatz et al., 2022). In the case of ARR for upper thermal tolerance, ARR = [(highest CT_max_ – lowest CT_max_) /Δ°C]. Then it provides a metric of thermal plasticity capturing acclimation capacity, thus allowing standardized comparisons between populations and critical thermal limits, (e.g., whether beneficial acclimation is greater for CT_min_ than CT_max_). During CT_min_ estimates at the lower acclimation temperatures (6 and 13°C), water often reached crystallization (exothermic freezing reaction) before the immobility endpoint was attained. Most of the tadpoles briefly exposed to the freezing point recovered activity within a few seconds and survived the 24‐h period, indicating resistance to these extreme cold temperatures, but the actual CT_min_ was inestimable (i.e., in these cases CT_min_ was lower [cooler] than the observed freezing point of water). Thus, in order to avoid bias associated with either biased sample or a biased CT_min_ estimates, we restricted the statistical analyses of CT_min_ acclimation scope to the 20–27°C range. All the statistical analyses were performed in R 3.6.1 (R Core Team, 2019).

## 3 RESULTS

**FIGURE 3:**
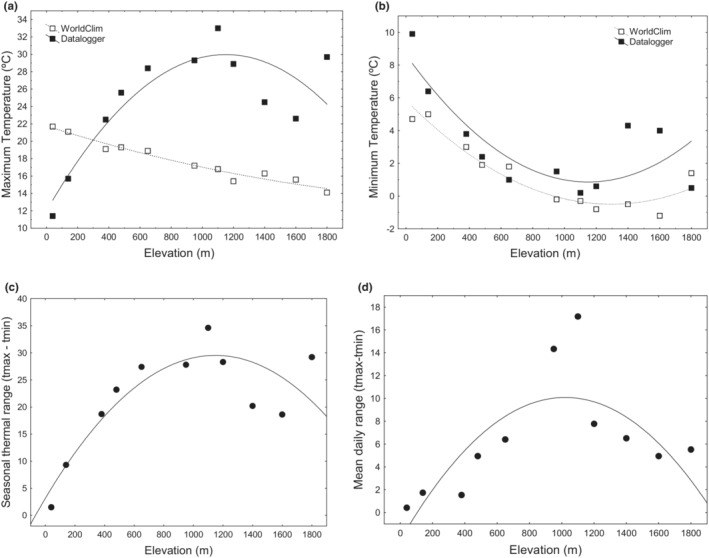
Elevational variation in: Absolute maximum (a) and minimum temperatures (b) using the WorldClim database (TMAX and TMIN), and microenvironmental pond datalogger information (tmax and tmin). Absolute seasonal temperature range (c) (st = tmax‐tmin) and mean daily temperature range (dr) (daily tmax‐tmin) (d), based in microenvironmental pond datalogger information. Thermal information corresponds only to the larval period for each studied location (see Figure 1b).

Pond minimum temperatures (TMIN, tmin) changed with altitude according to a quadratic function (Figure 3b; Table 2). However, for maximum temperatures we found a contrasting pattern of variation between macro‐ and microclimatic indicators along the elevation gradient (TMAX vs tmax, *t* = −3.1944, df = 19, *p* < .01; D = 0.64, *p* < .05). While TMAX monotonically decreased with altitude (*R* ^2^ = 0.97, *p* < .01; Figure 3a), tmax increased with elevation and peaked at mid‐altitude (*R* ^2^ = 0.77, *p* < .01; Figure 3a, Table 2). Similarly, both seasonal and diel temperature ranges increased with elevation and peaked at mid‐altitude populations (Table 2, Figure 3c,d).

**TABLE 2:** Results for the linear and quadratic regressions between temperature data, critical thermal limits elevation, warming tolerance (WT, wt) and cooling tolerance (CT, ct).

Model						
Dependent	Predictors	Equation	*F‐value*	df	*R* ^2^	*p‐value*
TMAX~	Elevation+Elevation^2	y = 21.76–0.006x + 9.25E‐7x^2	162.17	2,8	0.97	.0000001
tmax~	Elevation+Elevation^2	y = 12 + 3.12E‐2x‐1.36E‐05x^2^	13.77	2,8	0.77	<.01
TMIN~	Elevation+Elevation^2	y = 5.85–9.81E‐3x + 3.79E‐6x^2	49.44	2,8	0.93	.0026
tmin~	Elevation+Elevation^2	y = 8.63–1.35E‐2x + 5.89E‐6x^2	8.12	2,8	0.67	.012
SR~	Elevation+Elevation^2	y = 7.03 + 1.14E‐3x‐1.01E‐6x^2	5.81	2,8	0.59	.028
sr	Elevation+Elevation^2	y = 3.14 + 0.046x‐2.01E‐5x^2sr	66.68	2,8	0.74	.00001
dr~	Elevation+Elevation^2	y = −2.44 + 0.024x‐1.16E‐5x^2	15.71	2,8	0.60	.002
CT_max_~	Elevation	y = 36.7 + 1.92E‐4x	1.61	1,9	0.15	.236
CT_max_~	TMAX	y = 37.55–0.04x	0.99	1,9	0.10	.346
CT_max_~	tmax	y = 36.48 + 0.01x	1.06	1,9	0.11	.330
CT_max_~	SR	y = 38.62–0.26x	3.72	1,9	0.29	.086
CT_max_~	dr	y = 36.76 + 0.02x	0.44	1,9	0.05	.524
CT_min_~	Elevation	y = −1.62–1.5E‐4x	0.55	1,9	0.06	.477
CT_min_~	TMIN	y = −1.80 + 0.03x	0.25	1,9	0.03	.629
CT_min_~	tmin	y = −1.91 + 0.05x	1.69	1,9	0.16	.226
CT_min_~	SR	y = −3.77 + 0.29x	2.75	1,9	0.23	.132
CT_min_~	dr	y = −1.75–0.002x	0.004	1,9	<0.01	.951
WT~	Elevation	y = 15.38 + 4.18E‐3x	157.50	1,9	0.95	.000001
WT~	SR	y = 37.06–2.60x	6.64	1,9	0.42	.03
wt~	Elevation+Elevation^2	y = 24.63–3.08E‐2x + 1.34E‐5x^2	13.26	2,8	0.71	.003
wt~	dr	y = 20.34–1.47x	24.49	1,9	0.73	.0008
CT	Elevation+Elevation^2	y = 7.38–9.31e‐3x + 3.59E‐6x^2	24.36	2,8	0.86	.0004
CT	SR	y = 1.38 + 0.25x	0.05	1,9	0.01	.828
ct	Elevation+Elevation^2	y = 10.15–1.32E‐2x + 5.68E‐6x^2	8.17	2,8	0.67	.012
ct	dr	y = 8.70–0.71x	24.89	1,9	0.73	.0007

TMAX and TMIN (maximum and minimum temperatures from macroclimate); tmax and tmin (maximum and minimum temperatures from dataloggers; sr, seasonal temperature range (tmax‐tmin); dr, diel temperature range

A preliminary analysis revealed that tadpoles from populations between 900 and 1700 m were smaller than tadpoles from low elevation (<700 m) and the highest elevation (1800 m) (ANOVA; *F*_10,325_ = 57.26, *p* < .0001). In general, critical thermal limits were unaffected by tadpole size. The effect of tadpole mass on CT_max_ was not significant in any of the populations (Color: *p* = .07; all the rest *P*s >0.26). Tadpole mass has a negative effect on CT_min_ in Nueva (*F*_1,11_ = 8.91; *p* = .012), Pandecarmen (*F*_1,12_ = 8.10; *p* = .015), and Pandébano (*F*_1,14_ = 6.54; *p* = .023) (that is, larger tadpoles reached cooler temperatures), but not for Fana (*F*_1,14_ = 3.45; *p* = .084) and the rest of the populations (all *P*s >0.17).

**FIGURE 4:**
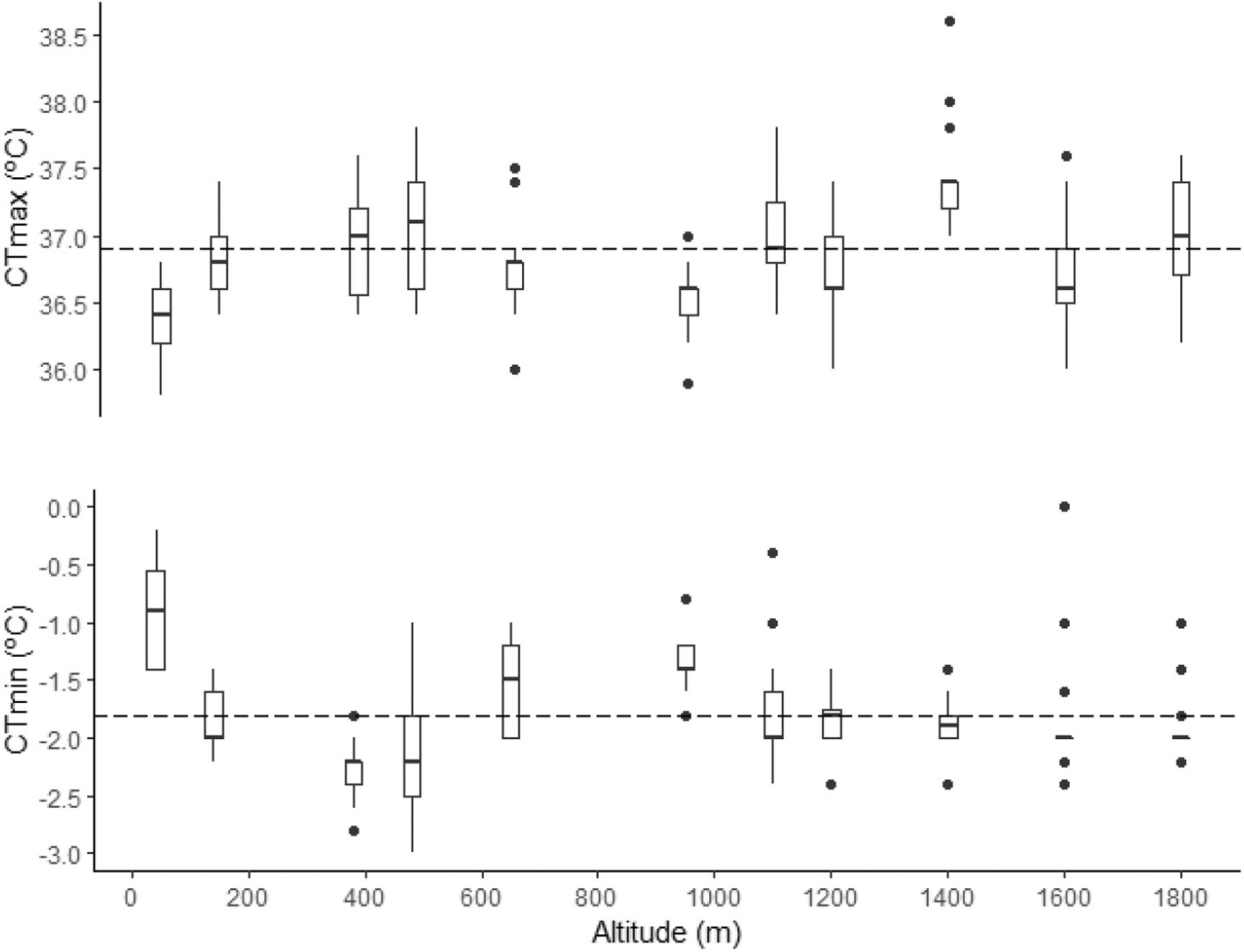
Boxplot showing the variation of thermal tolerance limits along the elevational gradient. The first and third quartiles (“hinges”) and the 95% confidence interval of the median (“notches”) are shown. The dashed line indicates the mean CT values of *R. parvipalmata* for the overall data, and dots placed past the line edges indicate outliers.

**FIGURE 5:**
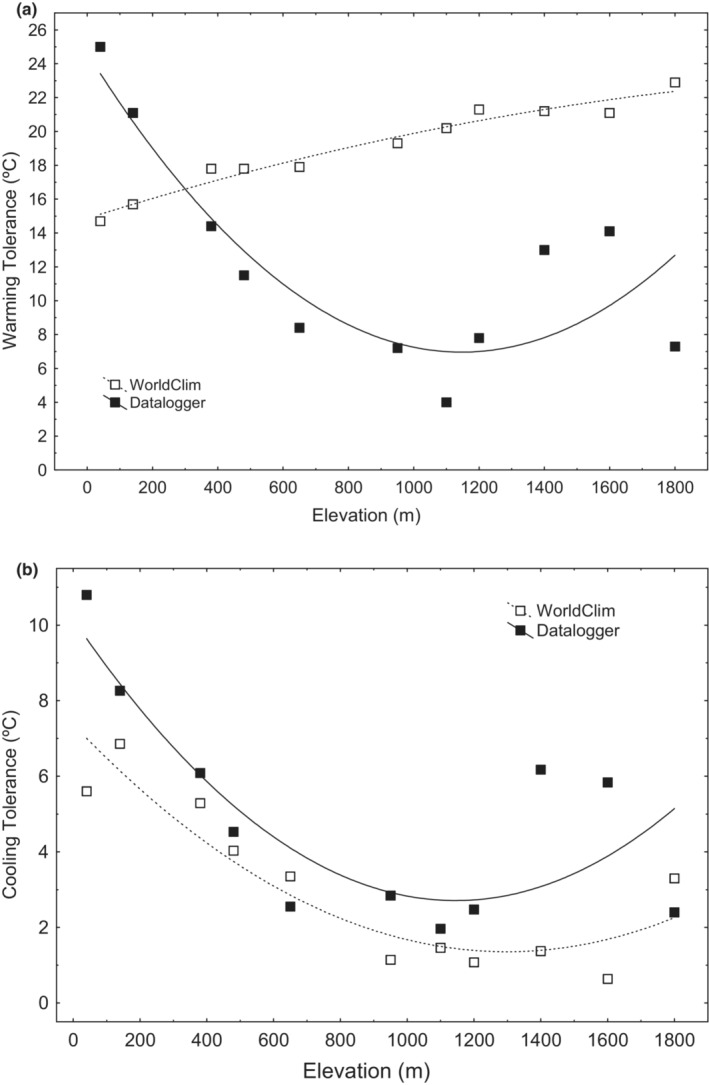
Estimates of Warming Tolerance: CT_max_ minus TMAX or tmax, for either WorldClim or microclimate pond maximum temperature estimates (datalogger), respectively (a), and Cooling Tolerances: TMIN or tmin, for either WorldClim or microclimate pond minimum temperatures (datalogger), respectively, minus CT_min_ (b), for 11 populations of *R. parvipalmata*, considering only the larval period. Triangles: estimates based on air temperature from WorldClim. Squares: estimates based on water temperatures registered in ponds with dataloggers.

Critical thermal limits significantly differed between populations (CT_max_, ANCOVA *F*_10,149_ = 8.83, *p* < .001; CT_*min*_, ANCOVA *F*_*10,149*_ = 15.39, *p* < .001) (Tables 1 and Table S1) although they were not affected by either elevation or any or macro‐ and microclimatic predictor (Figure 4; Table 2). Regarding vulnerability to heat stress, we also found contrasting differences between warming tolerance estimates based on macro‐ (WT; WorldClim) and micro‐climate data (wt) (*t* = 2.74, df = 10, *p* = .02; D = 0.82, *p* < .01). These differences were remarkable in two ways. First, WT was consistently higher than wt, except for two populations at the lowest elevation (Figure 5a). Second, WT increased linearly with altitude (*R* ^*2*^ = 0.95, *p* < .01), whereas wt showed a minimum at mid‐altitude sites (*R* ^2^ = 0.71, *p* < .01) (Table 2, Figure 5a). In contrast, cooling tolerances exhibit a non‐linear decrease with elevation using both micro and macro‐climatic estimators, Table 2, Figure 5b. Finally, both warming (wt) and cooling (ct) tolerances decrease with increasing daily temperature range (dr) (Table 2).

The overall value of neutral differentiation (F_ST_) of *R. parvipalmata* was 0.066 (95% CI: 0.058–0.075) and revealed significant genetic differentiation among the 11 populations (Table S8). Under the null hypothesis *c* = *h* ^2^, both CT_max_ and CT_min_ showed higher overall P_ST_ values than the upper confidence interval for F_ST_ (P_ST_ CT_max_, 95% CI: 0.12–0.21; P_ST_ CT_min_, 95% CI: 0.17–0.30) (Figure S1, Tables S9 and S10). However, the significance of this difference was not very robust, as the lower confidence estimate of P_ST_ overlaps with the upper limit of F_ST_ when *c*/*h* ^*2*^ = 0.51 for CT_max_, and when *c*/*h* ^*2*^ = 0.29 for CT_min_. The pairwise P_ST_ and F_ST_ matrices were not correlated for either CT_max_ or CT_min_ under the null hypothesis (*c* = *h* ^2^) (CT_max_ *r* = 0.066, *p* = .40; CT_min_ *r* = −0.1695, *p* = .75).

**FIGURE 6:**
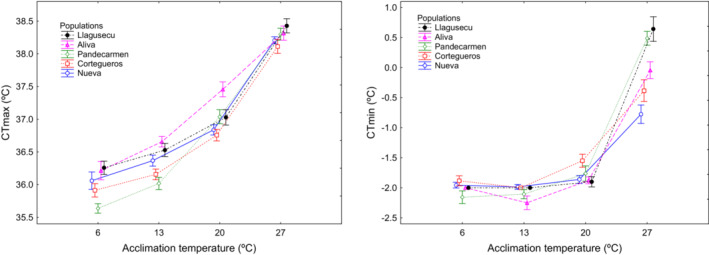
Phenotypic plasticity of critical thermal limits, CT_max_ (left) and CT_min_ (right), in five Rana parvipalmata populations: Nueva (140 m), Cortegueros (650 m), Pandecarmen (1106 m), Aliva (1418 m) and Llagusecu (1835 m), acclimated to different constant temperatures. Data of plasticity in CT_min_ at 6°C and 13°C were excluded from statistical analyses because in most occasions water was frozen once it reaches crystallization point. Here, they are shown as an upper bound for CT_min_ (‘true’ CT_min_ were below the values showed for 6°C and 13°C acclimation temperature). Values for each population / acclimation treatment are Means ±1 SE.

We found significant divergence among populations in the level of plasticity of CT_max_ and CT_min_ to variation in acclimation temperature (population × acclimation interaction; Table 3). Acclimation to warm temperatures resulted in higher CT_max_ and CT_min_ (Table 4, Figure 6). Mid and high‐altitude populations showed the highest phenotypic plasticity for CT_min_ (ARR; mean ± 1SD, 0.32 ± 0.05, *n* = 3, Table 4), being twice greater in magnitude when compared with the ARRs of lowland populations (mean ± 1SD, 0.16 ± 0.01, *n* = 2; Table 4). Greater ARR indexes for CT_max_ were obtained for the mid‐ elevation populations (Table 4).

**TABLE 3:** Two‐way ANOVA for changes in critical thermal limits (CT_max_ and CT_min_) in five populations acclimated to several temperatures (6, 13, 20°C and 27°C for CT_max_; 20 and 27°C for CT_min_)

	CT_max_				
	Df	Sum Sq	Mean Sq	*F* value	Pr(>F)
Population	4	6.298	1.574	10.957	<0.001
Acclimation temperature	3	222.322	74.107	515.7394	<0.001
Population × temperature	12	4.382	0.365	2.5413	0.003
Residuals	274	39.371	0.144		

	CT_min_				
	Df	Sum Sq	Mean Sq	F value	Pr(>F)
Population	4	10.342	2.585	11.087	<0.001
Acclimation temperature	1	116.51	116.51	499.652	<0.001
Population × temperature	4	11.105	2.776	11.906	<0.001
Residuals	130	30.314	0.2333		

**TABLE 4:** Variation in the thermal physiology limits in five populations of *R. parvipalmata*, acclimated to constant temperatures (CT_max_: 6, 13, 20, 27°C; CT_min_: 20, 27 C). Plasticity for CT_max_ is estimated as the difference between mean CT_max_ values at the 27 and 6 C acclimation treatments, whereas in CT_min_ is estimated as the difference between mean CT_min_ values at the 27 and 20 C acclimation treatments. For CT_max_, ARR = [(highest CT_max_ – lowest CT_max_) /Δ°C]. For CT_min_, ARR = [(CT_min_ 27°C–CT_min_ 20°C)/Δ°C].

CT_max_ (°C)															
Acclimation	Nueva			Cortegueros			Pandecarmen			Aliva			Llagusecu		
	*N*	$\bar{X}$	*SE*	*N*	$\bar{X}$	*SE*	*N*	$\bar{X}$	*SE*	*N*	$\bar{X}$	*SE*	*N*	$\bar{X}$	*SE*
6 C	11	36.06	0.13	16	35.91	0.10	17	35.64	0.07	15	36.21	0.14	14	36.26	0.10
13 C	12	36.37	0.08	16	36.16	0.09	17	36.02	0.09	14	36.65	0.08	14	36.53	0.10
20 C	16	36.84	0.08	16	36.76	0.09	16	37.04	0.11	14	37.46	0.11	14	37.03	0.12
27 C	16	38.21	0.05	16	38.11	0.11	17	38.30	0.09	12	38.32	0.11	14	38.43	0.11
Plasticity		2.15			2.20			2.70			2.11			2.17	
ARR 6–27°C	0.102			0.105			0.127			0.100			0.103		
ARR 6°–20°C	0.051			0.064			0.1			0.093			0.05		
ARR 20–27°C	0.196			0.194			0.18			0.123			0.2		

CT_min_ (°C)															
	Nueva			Cortegueros			Pandecarmen			Aliva			Llagusecu		
Acclimation	*N*	$\bar{X}$	SE	*N*	$\bar{X}$	SE	*N*	$\bar{X}$	SE	*N*	$\bar{X}$	SE	*N*	$\bar{X}$	SE
20°C	13	−1.86	0.06	12	−1.55	0.11	16	−1.76	0.13	16	−1.87	0.06	14	−1.90	0.08
27°C	12	−0.77	0.15	12	−0.38	0.18	17	0.49	0.11	14	−0.04	0.14	14	0.64	0.20
Plasticity		1.09			1.17			2.25			1.83			2.54	
ARR (20–27°C)	0.156			0.167			0.321			0.261			0.363		

## 4 DISCUSSION

Thermal limits (CT_max_, CT_min_) of tadpoles varied between *Rana parvipalmata* populations across the elevation gradient. However, CT_max_ and CT_min_ did not correlate with elevation nor with any of the macro‐ and microclimate predictors, suggesting niche conservatism. We did find some indications of directional selection as the divergence in thermal physiology limits (P_ST_) tended to be slightly greater than the neutral differences among populations (F_ST_). Yet, since the differentiation matrices F_ST_‐P_ST_ were not correlated, and thermal physiology limits were not related to elevation or any of the environmental variables, our data did not support the hypothesis of local adaptation in thermal limits. The neutral genetic differentiation observed among the studied populations of *R. parvipalamata* (F_ST_ = 0.066) is weak but well within the values of global F_ST_ reported for other amphibians over a wide spectrum of geographical scales and structured systems (Seppä & Laurila, 1999: 0.068; Luquet et al., 2015: 0.046, 0.024, and 0.011; Lenhardt et al., 2017: 0.041, 0.0159, 0.0215 and 0.0987).

The prevalence of intraspecific adaptive clinal variation with elevation in thermal traits is still a contentious topic in current literature. Although several studies have found support for this pattern in both Critical Thermal Limits (Bishop et al., 2017; Klok & Chown, 2003; Miller & Packard, 1977; Sørensen et al., 2005) with greater variation in CT_min_ than CT_max_ (Gilbert & Miles, 2019; Muñoz et al., 2014), many others did not (Buckley et al., 2013; Gvoždík & Castilla, 2001; Slatyer et al., 2016; Slatyer & Schoville, 2016; Senior et al., 2019; Slatyer et al., 2019; Tonione et al., 2020; Enriquez‐Urzelai et al., 2018, 2020). The lack of clinal variation in *R. parvipalmata* contrasts with the observation of local adaptation in larval life history traits of other temperate amphibians in seasonal thermal gradients (Berven et al., 1979; Luquet et al., 2015; Richter‐Boix et al., 2015), including the closely related *Rana temporaria* (Laugen et al., 2003; Lind et al., 2011; Muir et al., 2014). However, our findings were consistent with the absence of elevational variation in thermal sensitivity of locomotion and thermotolerance reported for post‐metamorphic and adults of *Rana parvipalmata* in the same study system (Enriquez‐Urzelai et al., 2018, 2020). In that case, the terrestrial stages of amphibians can thermoregulate via micro‐habitat selection or activity timing, sheltering underground in crevices and rodent burrows to avoid peak temperatures (Enriquez‐Urzelai et al., 2020). This is parallel to the known Bogert effect in many ecotherms, (Bogert, 1949; Buckley et al., 2015; Farallo et al., 2018; Huey et al., 2003; Muñoz, 2022; Muñoz & Losos, 2018). However, the scope for behavioral thermoregulation in water is much more limited than in the terrestrial environment due to its high specific heat capacity and conductivity that reduces spatial thermal heterogeneity. Amphibian tadpoles, although able to thermoregulate (Hutchison & Dupré, 1992), can be exposed to unavoidably thermal stress, particularly in sunlit ponds without canopy cover (Duarte et al., 2012). Exposure to sunlight is prevalent in the breeding habitats of mid‐ and high‐elevation populations of *R. parvipalmata*, which are exposed to relatively stressful high temperatures with warming tolerances <8°C (Figure 5a) and a wide range of both seasonal and short‐term thermal variation (Figure 3d).

Shifts in the reproductive period and microclimatic conditions that are conditioned by elevation may dampen temperature changes along the gradient and prevent the expected linear decrease in both critical thermal limits. Therefore, thermal conservatism in tolerance limits together the absence of local adaptation may simply be a consequence of a coupled variation of reproductive timing with elevation (the “elevation‐time axis” for temperature variation, in contrast with the elevation axis). In montane areas, altitudinal variation in the timing of reproduction seems to be constrained by hydroperiod rather than temperature itself, with higher altitude populations delaying breeding until snow melting (Álvarez et al., 2012; Corn, 2003). In addition, the time of spawning may be genetically determined in mountain populations (Álvarez et al., 2012; Phillimore et al., 2010; Wilczek et al., 2010). In this sense, it appears that during warm winters, when early snow melting occurs, frogs still delay reproduction until a threshold time is reached (Álvarez et al., 2012).

Phenotypic plasticity of thermal limits matched the observed variability in temperature through the elevation gradient. Populations from mid and high‐elevations showed higher levels of plasticity than low‐elevation populations, especially for CT_min_. These populations, particularly those from mid‐elevation, are also exposed to greater seasonal and daily thermal ranges, which supports the idea that phenotypic plasticity in critical thermal limits can be a response to the increased environment thermal variability. Similar adaptive plasticity in thermal tolerances are shown by populations of two toad species exposed to more variable climates (Alveal‐Riquelme et al., 2016; McCann et al., 2018). Furthermore, since mid‐elevation populations showed the lowest warming and cooling tolerances, and both thermal limits showed no clear clines, our data suggest the existence of a trade‐off between phenotypic plasticity and tolerance to environmental temperatures (Stillman, 2003). Acclimation to low temperatures allowed tadpoles to achieve tolerance to extreme cold beyond the physical freezing point of water. The common frogs are among the most cold‐tolerant amphibians of Europe (Gutiérrez‐Pesquera et al., 2016; see also Enriquez‐Urzelai et al., 2020) and, in northern Iberia, likely expanded during the cold glacial cycles (Dufresnes et al., 2020). Thus, it is possible that these extremely low CT_min_ are remnants of adaption to environmental conditions from that period, resulting in no current micro‐geographic adaptive differentiation along elevation gradients. This hypothesis of ‘evolutionary anachronism’ (Janzen & Martin, 1982; see also Qu & Wiens, 2020, Moreira et al., 2021) was supported by our finding that lowland populations showed extreme cold tolerances (e.g., Color, 380 m) although they presented the higher minimum temperature recorded.

The contrasting estimates of warming tolerances derived from macro‐ and microclimate data highlight the importance of monitoring the microhabitat when assessing vulnerability to global warming (Baudier et al., 2015; Gutiérrez‐Pesquera et al., 2016; Katzenberger et al., 2018; Enriquez‐Urzelai, Kearney, et al., 2019; Pintanel et al., 2019, 2022; see also Sunday et al., 2014). Warming tolerances estimated from macroclimate data supported the elevational thermal vulnerability hypothesis (risk of heat stress at low elevations and risk of cold stress at high elevations) (Pintanel et al., 2019, 2022; Sunday et al., 2014). Cooling tolerance (CT and ct) and warming tolerance estimated with microclimate data (wt), showed that mid‐elevations populations are more likely to suffer both cold and heat acute stress than populations from low and high elevations. These deviations from the expected patterns arise from two factors. First, despite both CT_max_ and CT_min_ can vary between populations this variation was very weak (1.1 and 1.4°C for CT_max_ and CT_min_, respectively). Besides, there was no distinct pattern across the elevation gradient and neither CT_max_ nor CT_min_ were related to any of the macro‐ and microclimate data (see also Richter‐Boix et al., 2015; Schou et al., 2017; Enriquez‐Urzelai, 2018; Enriquez‐Urzelai et al., 2020). Second, because of such weak variation in CT_max_ and CT_min_, the elevation variation in warming and cooling tolerances matched the pattern of variation in environmental temperatures, with only minor influence of thermal physiology limits. Only TMAX, used to determine WT, varied with elevation as expected, being lower at high elevation populations. However, both the lower minimum temperatures (TMIN and tmin) and higher tmax were found in mid‐elevation populations and not at the high‐peak populations. This a priori unexpected outcome is likely the result of phenological shifts in these local populations and differences in habitat structure (i.e., canopy cover, topographical shadow) between low‐, mid‐, and high‐elevation wetlands. In the study area, the breeding habitat of lowland (below 500–700 m) common frogs consists of very small and shallow waters scattered on a rather humanized landscape (e.g., track pools, ditches, and less often small temporary ponds), and located in valley bottoms with dense canopy cover, which prevents direct beam solar radiation. In contrast, breeding habitats in mid‐altitude areas (700–1200 m) are small, shallow, temporary ponds, most often located on high plains and hills without canopy cover, and therefore, exposed to high levels of direct solar radiation and a low thermal buffering. Finally, although high altitude wetlands (1300–2100 m) can be affected by topographic shading, most often they lack canopy cover, which besides a thinner atmosphere leads to low thermal buffering. This, along with the change in reproductive phenology (the elevation‐time axis), may reduce the actual differences in temperatures experienced by larvae at different elevations.

Phenological shifts and microgeographic variation in habitat structure can determine the thermal regimes experienced by populations along mountain gradients (see Muñoz & Losos, 2018). Seemingly, frog populations have responded to natural selection on breeding phenology, likely due to low reproductive success of too late breeders (high risk of pond drying in late winter/spring) in the lowlands and both early and late breeders in medium and high elevation populations (high risk of crushing due to late snowfall, pond drying in summer, and time constraints for the year recruits). In turn, this pattern of temporal displacement with elevation has reduced the thermal differences between populations, thus hindering physiological evolution (see also Enríquez‐Urzelai et al., 2018; Muñoz, 2022).

## 5 CONCLUSIONS

Directional changes in reproductive phenology and phenotypic plasticity can block local thermal adaptation by lowering selective pressures for population differentiation, (consistent with niche conservatism hypothesis; Muñoz & Losos, 2018). Therefore, although “phenological buffering” may override thermal stress under current conditions, it could hinder long‐term adaptation to climate change, potentially compromising long‐term population sensitivity (Buckley et al., 2015; Enriquez‐Urzelai et al., 2018; Kearney et al., 2009). This agrees with the idea that phenotypic responses do not occur in selective vacuums, and therefore, any adjustment in one response can cause an evolutionary ripple in others (Muñoz, 2022). For instance, if the timing of breeding is under genetic control, rapid climate change could cause temporal mismatches between physiological traits and the new thermal conditions with still unknown consequences. Our previous work on *R. parvipalmata* using biophysical models of thermal exposure indicated that the risk of reaching body temperatures beyond the species' thermal tolerance is similar across elevations, but mountain populations can face the worst climatic scenario because of their narrow seasonal activity windows (Enriquez‐Urzelai et al., 2018, 2020) and conflicting selection on breeding phenology. Present results reinforce this view: mountain populations of *R. parvipalmata* are also the most vulnerable during the aquatic phase. Therefore, future research should focus on the genetic component of reproductive phenology, the physiological responses of mountain populations, and the effects of space–time covariations in biological processes that can determine how the species face climate change.

## AUTHOR CONTRIBUTIONS

**Luis Miguel Gutiérrez‐Pesquera:** Conceptualization (equal); data curation (lead); formal analysis (equal); investigation (equal); methodology (equal); writing – original draft (equal); writing – review and editing (equal). **Miguel Tejedo:** Conceptualization (equal); data curation (equal); formal analysis (equal); funding acquisition (equal); investigation (equal); methodology (equal); project administration (equal); resources (equal); software (equal); supervision (equal); validation (equal); visualization (equal); writing – original draft (equal); writing – review and editing (equal). **Agustín Camacho:** Conceptualization (equal); data curation (equal); formal analysis (equal); methodology (equal); software (equal); supervision (equal); validation (equal); writing – original draft (equal); writing – review and editing (equal). **Urtzi Enriquez‐Urzelai:** Conceptualization (equal); data curation (equal); formal analysis (equal); writing – original draft (equal); writing – review and editing (equal). **Marco Katzenberger:** Conceptualization (equal); data curation (equal); formal analysis (equal); writing – original draft (equal); writing – review and editing (equal). **Magdalena Choda:** Conceptualization (equal); data curation (equal); formal analysis (equal); writing – original draft (equal); writing – review and editing (equal). **Pol Pintanel:** Conceptualization (equal); data curation (equal); formal analysis (equal); methodology (equal); writing – original draft (equal); writing – review and editing (equal). **Alfredo G. Nicieza:** Conceptualization (equal); data curation (equal); formal analysis (equal); funding acquisition (equal); investigation (equal); methodology (equal); project administration (equal); resources (equal); software (equal); supervision (equal); validation (equal); visualization (equal); writing – original draft (equal); writing – review and editing (equal).

## FUNDING INFORMATION

Spanish Ministry of Science and Innovation (MICINN CGL2012‐40246‐C02‐01, CGL2012‐40246‐C02‐02, BES‐2010‐032912). Spanish Ministry of Economy, Industry and Competitiveness (MINECO CGL2017‐86924‐P). Spanish Ministry of Education and Science (AP2005‐0143).

## Supporting information


Appendix S1

## Data Availability

Data are deposited on Figshare (https://doi.org/10.6084/m9.figshare.21187024.v1).

## References

[ece39349-bib-0001] Álvarez, D. , Choda, M. , Viesca, L. , Cano, J. M. , Bañuelos, M. J. , Matsuba, C. , García, S. , & Nicieza, A. G. (2012). Variación genética adaptativa en gradientes altitudinales: Efectos sobre la viabilidad de poblaciones subdivididas en escenarios de cambio climático. In L. Ramírez & B. Asensio (Eds.), Proyectos de Investigación en parques nacionales: 2008–2011. Naturaleza y Parques Nacionales. Serie investigación en la red (pp. 125–150). Organismo Autónomo Parques Nacionales.

[ece39349-bib-0002] Alveal‐Riquelme, N. , Díaz‐Páez, H. , & Ortiz, J. C. (2016). Thermal tolerance in the Andean toad *Rhinella spinulosa* (Anura: Bufonidae) at three sites located along a latitudinal gradient in Chile. Journal of Thermal Biology, 60, 237–245. 10.1016/j.jtherbio.2016.07.019 27503738

[ece39349-bib-0003] Angilletta, M. J. J. (2009). Thermal adaptation: A theoretical and empirical synthesis. Oxford University Press.

[ece39349-bib-0004] Bachmann, J. C. , Jansen van Rensburg, A. , Cortazar‐Chinarro, M. , Laurila, A. , & Van Buskirk, J. (2020). Gene flow limits adaptation along steep environmental gradients. The American Naturalist, 195, E67–E86. 10.1086/707209 32097047

[ece39349-bib-0005] Bates, D. , Mächler, M. , Bolker, B. , & Walker, S. (2015). Fitting linear mixed‐effects models using lme4. Journal of Statistical Software, 67, 1–48. 10.18637/jss.v067.i01

[ece39349-bib-0006] Baudier, K. M. , Mudd, A. E. , Erickson, S. C. , & O'Donnell, S. (2015). Microhabitat and body size effects on heat tolerance: Implications for responses to climate change (army ants: Formicidae, Ecitoninae). The Journal of Animal Ecology, 84, 1322–1330. 10.1111/1365-2656.12388 26072696

[ece39349-bib-0007] Berven, K. A. , Gill, D. E. , & Smith‐Gill, S. J. (1979). Countergradient selection in the green frog, *Rana clamitans* . Evolution, 33, 609–623. 10.1111/j.1558-5646.1979.tb04714.x 28563934

[ece39349-bib-0008] Bishop, T. R. , Robertson, M. P. , Rensburg, B. J. , & Parr, C. L. (2017). Coping with the cold: Minimum temperatures and thermal tolerances dominate the ecology of mountain ants. Ecological Entomology, 42, 105–114. 10.1111/een.12364

[ece39349-bib-0009] Bogert, C. M. (1949). Thermoregulation in reptiles, a factor in evolution. Evolution, 3, 195–211. 10.1111/j.1558-5646.1949.tb00021.x 18138377

[ece39349-bib-0010] Bozinovic, F. , Calosi, P. , & Spicer, J. I. (2011). Physiological correlates of geographic range in animals. Annual Review of Ecology, Evolution, and Systematics, 42, 155–179. 10.1146/annurev-ecolsys-102710-145055

[ece39349-bib-0011] Brommer, J. E. (2011). Whither Pst? The approximation of Qst by Pst in evolutionary and conservation biology. Journal of Evolutionary Biology, 24, 1160–1168. 10.1111/j.1420-9101.2011.02268.x 21457173

[ece39349-bib-0012] Buckley, L. B. , Ehrenberger, J. C. , & Angilletta, M. J. (2015). Thermoregulatory behavior limits local adaptation of thermal niches and confers sensitivity to climate change. Functional Ecology, 29, 1038–1047. 10.1111/1365-2435.12406

[ece39349-bib-0013] Buckley, L. B. , & Huey, R. B. (2016). How extreme temperatures impact organisms and the evolution of their thermal tolerance. Integrative and Comparative Biology, 56, 98–109. 10.1093/icb/icw004 27126981

[ece39349-bib-0014] Buckley, L. B. , Miller, E. F. , & Kingsolver, J. G. (2013). Ectotherm thermal stress and specialization across altitude and latitude. Integrative and Comparative Biology, 53, 571–581. 10.1093/icb/ict026 23620253

[ece39349-bib-0015] Caillon, R. , Suppo, C. , Casas, J. , Arthur Woods, H. , & Pincebourde, S. (2014). Warming decreases thermal heterogeneity of leaf surfaces: Implications for behavioural thermoregulation by arthropods. Functional Ecology, 28, 1449–1458. 10.1111/1365-2435.12288

[ece39349-bib-0016] Camacho, A. , Rodrigues, M. T. , & Navas, C. (2015). Extreme operative temperatures are better descriptors of the thermal environment than mean temperatures. Journal of Thermal Biology, 49, 106–111. 10.1016/j.jtherbio.2015.02.007 25774033

[ece39349-bib-0017] Camacho‐Sánchez, M. , Velo‐Antón, G. , Hanson, J. O. , Veríssimo, A. , Martínez‐Solano, I. , Marques, A. , Moritz, C. , & Carvalho, S. B. (2020). Comparative assessment of range‐wide patterns of genetic diversity and structure with SNPs and microsatellites: A case study with Iberian amphibians. Ecology and Evolution, 19, 10353–10363. 10.1002/ece3.6670 PMC7548196 33072264

[ece39349-bib-0018] Chevin, L. M. , & Hoffmann, A. A. (2017). Evolution of phenotypic plasticity in extreme environments. Philosophical Transactions of the Royal Society B, 372, 20160138. 10.1098/rstb.2016.0138 PMC5434089 28483868

[ece39349-bib-0019] Chevin, L. M. , Lande, R. , & Mace, G. M. (2010). Adaptation, plasticity, and extinction in a changing environment: Towards a predictive theory. PLoS Biology, 8, e1000357. 10.1371/journal.pbio.1000357 20463950 PMC2864732

[ece39349-bib-0020] Choda, M. (2014). Genetic variation and local adaptations of Rana parvipalmata in the Cantabrian Mountains. PhD Thesis. Universidad de Oviedo.

[ece39349-bib-0021] Claussen, D. L. (1977). Thermal acclimation in ambystomatid salamanders. Comparative Biochemistry and Physiology Part A: Physiology, 58, 333–340. 10.1016/0300-9629(77)90150-5

[ece39349-bib-0022] Corn, P. S. (2003). Amphibian breeding and climate change: Importance of snow in the mountains. Conservation Biology, 17, 622–625. 10.1046/j.1523-1739.2003.02111.x

[ece39349-bib-0023] DeFaveri, J. , Viitaniemi, H. , Leder, E. , & Merilä, J. (2011). Characterizing genic and nongenic molecular markers: Comparison of microsatellites and SNPs. Molecular Ecology Resources, 13, 377–392. 10.1111/1755-0998.12071 23356957

[ece39349-bib-0024] Deutsch, C. A. , Tewksbury, J. J. , Huey, R. B. , Sheldon, K. S. , Ghalambor, C. K. , Haak, D. C. , & Martin, P. R. (2008). Impacts of climate warming on terrestrial ectotherms across latitude. Proceedings of the National Academy of Sciences of the United States of America, 105, 6668–6672. 10.1073/pnas.0709472105 18458348 PMC2373333

[ece39349-bib-0025] Diamond, S. E. , & Chick, L. D. (2018). The Janus of macrophysiology: Stronger effects of evolutionary history, but weaker effects of climate on upper thermal limits are reversed for lower thermal limits in ants. Current Zoology, 64, 223–230. 10.1093/cz/zox072 30402063 PMC5905527

[ece39349-bib-0026] Duarte, H. , Tejedo, M. , Katzenberguer, M. , Marangoni, F. , Baldo, D. , Beltrán, J. F. , Martí, D. A. , Richter‐Boix, A. , & Gonzalez‐Voyer, A. (2012). Can amphibians take the heat? Vulnerability to climate warming in subtropical and temperate larval amphibian communities. Global Change Biology, 18, 412–421. 10.1111/j.1365-2486.2011.02518.x

[ece39349-bib-0027] Dufresnes, C. , Nicieza, A. G. , Litvinchuk, S. N. , Rodrigues, N. , Jeffries, D. L. , Vences, M. , Perrin, N. , & Martínez‐Solano, Í. (2020). Are glacial refugia hotspots of speciation and cytonuclear discordances? Answers from the genomic phylogeography of Spanish common frogs. Molecular Ecology, 29, 986–1000. 10.1111/mec.15368 32012388

[ece39349-bib-0028] Endler, J. A. (1977). Geographic variation, speciation, and clines. Princeton University Press. 409931

[ece39349-bib-0029] Enriquez‐Urzelai, U. , Kearney, M. R. , Nicieza, A. G. , & Tingley, R. (2019). Integrating mechanistic and correlative niche models to unravel range‐limiting processes in a temperate amphibian. Global Change Biology, 25, 2633–2647. 10.1111/gcb.14673 31050846

[ece39349-bib-0030] Enriquez‐Urzelai, U. , Palacio, A. S. , Merino, N. M. , Sacco, M. , & Nicieza, A. G. (2018). Hindered and constrained: Limited potential for thermal adaptation in post‐metamorphic and adult *Rana parvipalmata* along elevational gradients. Journal of Evolutionary Biology, 31, 1852–1862. 10.1111/jeb.13380 30256481

[ece39349-bib-0031] Enriquez‐Urzelai, U. , Sacco, M. , Palacio, A. S. , Pintanel, P. , Tejedo, M. , & Nicieza, A. G. (2019). Ontogenetic reduction in thermal tolerance is not alleviated by earlier developmental acclimation in *Rana temporaria* . Oecologia, 189, 385–394. 10.1007/s00442-019-04342-y 30694384

[ece39349-bib-0032] Enriquez‐Urzelai, U. , Tingley, R. , Kearney, M. R. , Sacco, M. , Palacio, A. S. , Tejedo, M. , & Nicieza, A. G. (2020). The roles of acclimation and behaviour in buffering climate change impacts along elevational gradients. Journal of Animal Ecology, 89, 1722–1734. 10.1111/1365-2656.13222 32221971

[ece39349-bib-0033] Farallo, V. R. , Muñoz, M. M. , Uyeda, J. C. , & Miles, D. B. (2020). Scaling between macro‐ to microscale climatic data reveals strong phylogenetic inertia in niche evolution in plethodontid salamanders. Evolution, 74, 799–991. 10.1111/evo.13959 32190909

[ece39349-bib-0034] Farallo, V. R. , Wier, R. , & Miles, D. B. (2018). The Bogert effect revisited: Salamander regulatory behaviors are differently constrained by time and space. Ecology and Evolution, 8, 11522–11532. 10.1002/ece3.4590 30598753 PMC6303756

[ece39349-bib-0035] Feder, M. E. , & Hofmann, G. E. (1999). Heat‐shock proteins, molecular chaperones, and the stress response: Evolutionary and ecological physiology. Annual Review of Physiology, 61, 243–282. 10.1146/annurev.physiol.61.1.243 10099689

[ece39349-bib-0036] Fick, S. E. , & Hijmans, R. J. (2017). WorldClim 2: New 1‐km spatial resolution climate surfaces for global land areas. International Journal of Climatology, 37, 4302–4315. 10.1002/joc.5086

[ece39349-bib-0037] Floyd, R. B. (1983). Ontogenetic change in the temperature tolerance of larval Bufo mari‐nus (Anura: Bufonidae). Comparative Biochemistry and Physiology, 75A, 267–271. 10.1016/0300-9629(83)90081-6

[ece39349-bib-0038] Garland, T. , Adolph, S. C. , & Adolph, C. (1991). Physiological differentiation of vertebrate populations. Annual Review of Ecology and Systematics, 22, 193–228. 10.1146/annurev.es.22.110191.001205

[ece39349-bib-0039] Gilbert, A. L. , & Miles, D. B. (2019). Spatiotemporal variation in thermal niches suggests lability rather than conservatism of thermal physiology along an environmental gradient. Biological Journal of the Linnean Society, 128, 263–277. 10.1093/biolinnean/blz093

[ece39349-bib-0040] Gilchrist, G. W. (1995). Specialists and generalists in changing environments. I. Fitness landscapes of thermal sensitivity. The American Naturalist, 146, 252–270. 10.1086/285797

[ece39349-bib-0041] Gosner, K. L. (1960). A simplified table for staging anuran embryos and larvae with notes on identification. Herpetologica, 16, 183–190. 10.2307/3890061

[ece39349-bib-0042] Goudet, J. (2002). FSTAT, a program to estimate and test gene diversities and fixation indices.

[ece39349-bib-0043] Gunderson, A. R. , & Stillman, J. H. (2015). Plasticity in thermal tolerance has limited potential to buffer ectotherms from global warming. Proc. R. Soc. London B Biol. Sci., 282, 20150401. 10.1098/rspb.2015.0401 PMC4455808 25994676

[ece39349-bib-0044] Guo, S. W. , & Thompson, E. A. (1992). Performing the exact test of Hardy‐Weinberg proportion for multiple alleles. Biometrics, 48, 361–372. 10.2307/2532296 1637966

[ece39349-bib-0045] Gutiérrez‐Pesquera, L. M. , Tejedo, M. , Olalla‐Tárraga, M. Á. , Duarte, H. , Nicieza, A. , & Solé, M. (2016). Testing the climate variability hypothesis in thermal tolerance limits of tropical and temperate tadpoles. Journal of Biogeography, 43, 1166–1178. 10.1111/jbi.12700

[ece39349-bib-0046] Gvoždík, L. , & Castilla, A. M. (2001). A comparative study of preferred body temperatures and critical thermal tolerance limits among populations of *Zootoca vivipara* (Squamata: Lacertidae) along an altitudinal gradient. Journal of Herpetology, 35, 486–492. 10.2307/1565967

[ece39349-bib-0047] Habary, A. , Johansen, J. L. , Nay, T. J. , Steffensen, J. F. , & Rummer, J. L. (2017). Adapt, move or die—how will tropical coral reef fishes cope with ocean warming? Global Change Biology, 23, 566–577. 10.1111/gcb.13488 27593976

[ece39349-bib-0048] Helmuth, B. (2009). From cells to coastlines: How can we use physiology to forecast the impacts of climate change? The Journal of Experimental Biology, 212, 53–60. 10.1242/jeb.023861 19251989

[ece39349-bib-0049] Hijmans, R. J. , Cameron, S. E. , Parra, J. L. , Jones, P. G. , & Jarvis, A. (2005). Very high resolution interpolated climate surfaces of global land areas. International Journal of Climatology, 25, 1965–1978. 10.1002/joc.1276

[ece39349-bib-0050] Hijmans, R. J. & van Etten, J. (2014). Raster: Geographic data analysis and modeling. https://CRAN.R‐project.org/package=raster.

[ece39349-bib-0051] Hodkinson, I. D. (2005). Terrestrial insects along elevation gradients: Species and community responses to altitude. Biological Reviews, 80, 489–513. 10.1017/S1464793105006767 16094810

[ece39349-bib-0052] Hoffmann, A. A. , Chown, S. L. , & Clusella‐Trullas, S. (2013). Upper thermal limits in terrestrial ectotherms: How constrained are they? Functional Ecology, 27, 934–949. 10.1111/j.1365-2435.2012.02036.x

[ece39349-bib-0053] Hoffmann, A. A. , & Sgrò, C. M. (2011). Climate change and evolutionary adaptation. Nature, 470, 479–485. 10.1038/nature09670 21350480

[ece39349-bib-0054] Huey, R. B. , Hertz, P. E. , & Sinervo, B. (2003). Behavioral drive versus behavioral inertia in evolution: A null model approach. The American Naturalist, 161, 357–366. 10.1086/346135 12699218

[ece39349-bib-0055] Huey, R. B. , Kearney, M. R. , Krockenberger, A. , Holtum, J. A. M. , Jess, M. , & Williams, S. E. (2012). Predicting organismal vulnerability to climate warming: roles of behaviour, physiology and adaptation. Philosophical Transactions of the Royal Society B, 367, 1665–1679. 10.1098/rstb.2012.0005 PMC3350654 22566674

[ece39349-bib-0056] Hutchison, V. H. (1961). Critical thermal maxima in salamanders. Physiological Zoology, 34, 92–125.

[ece39349-bib-0057] Hutchison, V. H. , & Dupré, R. K. (1992). Thermoregulation. In M. E. Feder & W. M. Burggren (Eds.), Environmental Physiology of the Amphibians (pp. 206–249). The University of Chicago Press.

[ece39349-bib-0058] IPCC . (2014). Climate Change 2014: Synthesis Report. Contribution of Working Groups I, II and III to the Fifth Assessment Report of the Intergovernmental Panel on Climate Change. IPCC.

[ece39349-bib-0059] Janzen, D. H. (1967). Why mountain passes are higher in the tropics. American Naturalist, 101, 233–247. 10.1086/282487

[ece39349-bib-0060] Janzen, D. H. , & Martin, P. S. (1982). Neotropical anachronisms: The fruits the gomphotheres ate. Science, 215, 19–27. 10.1126/science.215.4528.19 17790450

[ece39349-bib-0061] Katzenberger, M. , Hammond, J. , Tejedo, M. , & Relyea, R. (2018). Source of environmental data and warming tolerance estimation in six species of North American larval anurans. Journal of Thermal Biology, 76, 171–178. 10.1016/j.jtherbio.2018.07.005 30143292

[ece39349-bib-0062] Kearney, M. , & Porter, W. P. (2017). NicheMapR – an R package for biophysical modelling: The microclimate model. Ecography, 40, 664–674. 10.1111/ecog.02360

[ece39349-bib-0063] Kearney, M. , & Porter, W. P. (2020). NicheMapR – an R package for biophysical modelling: The ectotherm and Dynamic Energy Budget models. Ecography, 43, 85–93. 10.1111/ecog.04680

[ece39349-bib-0064] Kearney, M. , Shine, R. , & Porter, W. P. (2009). The potential for behavioral thermoregulation to buffer “cold‐blooded” animals against climate warming. Proceedings of the National Academy of Sciences of the United States of America, 106, 3835–3840. 10.1073/pnas.0808913106 19234117 PMC2656166

[ece39349-bib-0065] Klok, C. J. , & Chown, S. L. (2003). Resistance to temperature extremes in sub‐Antarctic weevils: Interspecific variation, population differentiation and acclimation. Biological Journal of the Linnean Society, 78, 401–414. 10.1046/j.1095-8312.2003.00154.x

[ece39349-bib-0066] Laugen, A. T. , Laurila, A. , Räsänen, K. , & Merilä, J. (2003). Latitudinal countergradient variation in the common frog (*Rana temporaria*) development rates – evidence for local adaptation. Journal of Evolutionary Biology, 16, 996–1005. 10.1046/j.1420-9101.2003.00560.x 14635915

[ece39349-bib-0067] Leinonen, T. , Cano, J. M. , Mäkinen, H. , & Merilä, J. (2006). Contrasting patterns of body shape and neutral genetic divergence in marine and lake populations of threespine sticklebacks. Journal of Evolutionary Biology, 19, 1803–1812. 10.1111/j.1420-9101.2006.01182.x 17040377

[ece39349-bib-0068] Leinonen, T. , O'Hara, R. B. , Cano, J. M. , & Merilä, J. (2008). Comparative studies of quantitative trait and neutral marker divergence: A meta‐analysis. Journal of Evolutionary Biology, 21, 1–17. 10.1111/j.1420-9101.2007.01445.x 18028355

[ece39349-bib-0069] Lemopoulos, A. , Prokkola, J. M. , Uusi‐Heikkilä, S. , Vasemägi, A. , Huusko, A. , Hyvärinen, P. , Koljonen, M.‐L. , Koskiniemi, J. , & Vainikka, A. (2019). Comparing RADseq and microsatellites for estimating genetic diversity and relatedness — Implications for brown trout conservation. Ecology and Evolution, 9, 2106–2120. 10.1002/ece3.4905 30847096 PMC6392366

[ece39349-bib-0070] Lenhardt, P. P. , Brühl, C. A. , Leeb, C. , & Theissinger, K. (2017). Amphibian population genetics in agricultural landscapes: Does viniculture drive the population structuring of the European common frog (*Rana temporaria*)? PeerJ, 5, e3520. 10.7717/peerj.3520 28713651 PMC5508807

[ece39349-bib-0071] Levins, R. (1969). Thermal acclimation and heat resistance in *Drosophila* species. The American Naturalist, 103, 483–499. 10.1086/282616

[ece39349-bib-0072] Lind, M. I. , Ingvarsson, P. K. , Johansson, H. , Hall, D. , & Johansson, F. (2011). Gene flow and selection on phenotypic plasticity in an Island system of *Rana temporaria* . Evolution, 65, 684–697. 10.1111/j.1558-5646.2010.01122.x 20825480

[ece39349-bib-0073] Luquet, E. , Léna, J. P. , Miaud, C. , & Plénet, S. (2015). Phenotypic divergence of the common toad (Bufo bufo) along an altitudinal gradient: Evidence for local adaptation. Heredity, 114, 69–79. 10.1038/hdy.2014.71 25074572 PMC4815602

[ece39349-bib-0074] Lutterschmidt, W. I. , & Hutchison, V. H. (1997a). The critical thermal maximum: Data to support the onset of spasms as the definitive end point. Canadian Journal of Zoology, 75, 1553–1560. 10.1139/z97-782

[ece39349-bib-0075] Lutterschmidt, W. I. , & Hutchison, V. H. (1997b). The critical thermal maximum: History and critique. Canadian Journal of Zoology, 75, 1561–1574. 10.1139/z97-783

[ece39349-bib-0076] Mallard, F. , Nolte, V. , & Schlötterer, C. (2020). The evolution of phenotypic plasticity in response to temperature stress. Genome Biology and Evolution, 12, 2429–2440. 10.1093/gbe/evaa206 33022043 PMC7846148

[ece39349-bib-0077] McCann, S. M. , Kosmala, G. K. , Greenlees, M. J. , & Shine, R. (2018). Physiological plasticity in a successful invader: Rapid acclimation to cold occurs only in cool‐climate populations of cane toads (*Rhinella marina*). Conservation Physiology, 6, cox 072. 10.1093/conphys/cox072 PMC5786208 29399360

[ece39349-bib-0078] Miller, K. , & Packard, G. C. (1977). An altitudinal cline in critical thermal maxima of Chorus frogs (*Pseudacris triseriata*). The American Naturalist, 111, 267–277. 10.1086/283159

[ece39349-bib-0079] Moreira, M. O. , Qu, Y.‐F. , & Wiens, J. J. (2021). Large‐scale evolution of body temperatures in land vertebrates. Evolution Letters, 5, 484–494. 10.1002/evl3.249 34621535 PMC8484719

[ece39349-bib-0080] Muir, A. P. , Biek, R. , Thomas, R. , & Mable, B. K. (2014). Local adaptation with high gene flow: Temperature parameters drive adaptation to altitude in the common frog (*Rana temporaria*). Molecular Ecology, 23, 561–574. 10.1111/mec.12624 24330274 PMC4285318

[ece39349-bib-0081] Muñoz, M. M. (2022). The Bogert effect, a factor in evolution. Evolution, 76, 49–66. 10.1111/evo.14388 34676550

[ece39349-bib-0082] Muñoz, M. M. , & Losos, J. B. (2018). Thermoregulation simultaneously promotes and forestalls evolution in a tropical lizard. American Naturalist, 191, E15–E26. 10.1086/694779 29244559

[ece39349-bib-0083] Muñoz, M. M. , Stimola, M. A. , Algar, A. C. , Conover, A. , Rodriguez, A. J. , Landestoy, M. A. , Bakken, G. S. , & Losos, J. B. (2014). Evolutionary stasis and lability in thermal physiology in a group of tropical lizards. Proceedings of the Royal Society, 281, 20132433. 10.1098/rspb.2013.2433 PMC3906933 24430845

[ece39349-bib-0084] Navas, C. A. , Carvajalino‐Fernández, J. M. , Saboy‐Acosta, L. P. , Rueda‐Solano, L. A. , & Carvajalino‐Fernández, M. A. (2013). The body temperature of active amphibians along a tropical elevation gradient: Patterns of mean and variance and inference from environmental data. Functional Ecology, 27, 1145–1154. 10.1111/1365-2435.12106

[ece39349-bib-0085] Oksanen, J. , Blanchet, F. G. , Friendly, M. , Kindt, R. , Legendre, P. , McGlinn, D. , Minchin, P. R. , O’Hara, R. B. , Simpson, G. L. , Solymos, P. , Stevens, M. H. H. , Szoecs, E. , & Wagner, H. (2018). Package 'vegan': Community Ecology Package. R package Version 2.5‐2. https://CRAN.R‐project.org/package=vegan

[ece39349-bib-0086] Oosterhout, C. , Hutchinson, W. F. , Wills, D. P. M. , & Shipley, P. (2004). MICRO‐CHECKER: Software for identifying and correcting genotyping errors in microsatellite data. Molecular Ecology Notes, 4, 535–538. 10.1111/j.1471-8286.2004.00684.x

[ece39349-bib-0087] Overgaard, J. , Kearney, M. R. , & Hoffmann, A. A. (2014). Sensitivity to thermal extremes in Australian Drosophila implies similar impacts of climate change on the distribution of widespread and tropical species. Global Change Biology, 20, 1738–1750. 10.1111/gcb.12521 24549716

[ece39349-bib-0088] Pacifici, M. , Foden, W. B. , Visconti, P. , Watson, J. E. M. , Butchart, S. H. M. , Kovacs, K. M. , Scheffers, B. R. , Hole, D. G. , Martin, T. G. , Akçakaya, H. R. , Corlett, R. T. , Huntley, B. , Bickford, D. , Carr, J. A. , Hoffmann, A. A. , Midgley, G. F. , Pearce‐Kelly, P. , Pearson, R. G. , Williams, S. E. , … Rondinini, C. (2015). Assessing species vulnerability to climate change. Nature Climate Change, 5, 215–224. 10.1038/nclimate2448

[ece39349-bib-0089] Parmesan, C. (2006). Ecological and evolutionary responses to recent climate change. Annual Review of Ecology, Evolution, and Systematics, 37, 637–669. 10.1146/annurev.ecolsys.37.091305.110100

[ece39349-bib-0090] Phillimore, A. B. , Hadfield, J. D. , Jones, O. R. , & Smithers, R. J. (2010). Differences in spawning date between populations of common frog reveal local adaptation. Proceedings of the National Academy of Sciences of the United States of America, 107, 8292–8297. 10.1073/pnas.0913792107 20404185 PMC2889515

[ece39349-bib-0091] Pidancier, N. , Miquel, C. , & Miaud, C. (2003). Buccal swabs as a non‐destructive tissue sampling method for DNA analysis in amphibians. Herpetological Journal, 13, 175–178. https://www.thebhs.org/publications/the‐herpetological‐journal/volume‐13‐number‐4‐october‐2003/1731‐03‐buccal‐swabs‐as‐a‐non‐destructive‐tissue‐sampling‐method‐for‐dna‐analysis‐in‐amphibians

[ece39349-bib-0092] Pincebourde, S. , Murdock, C. C. , Vickers, M. , & Sears, M. W. (2016). Fine‐scale microclimatic variation can shape the responses of organisms to global change in both natural and urban environments. Integrative and Comparative Biology, 56, 45–61. 10.1093/icb/icw016 27107292

[ece39349-bib-0093] Pintanel, P. , Tejedo, M. , Merino‐Viteri, A. , Almeida‐Reinoso, F. , Salinas‐Ivanenko, S. , López‐Rosero, A. C. , Llorente, G. A. , & Gutiérrez‐Pesquera, L. M. (2022). Elevational and local climate variability predicts thermal breadth of mountain tropical tadpoles. Ecography, 2022, e05906. 10.1111/ecog.05906

[ece39349-bib-0094] Pintanel, P. , Tejedo, M. , Ron, S. R. , Llorente, G. A. , & Merino‐Viteri, A. (2019). Elevational and microclimatic drivers of thermal tolerance in Andean Pristimantis frogs. Journal of Biogeography, 46, 1664–1675. 10.1111/jbi.13596

[ece39349-bib-0095] Polato, N. R. , Gill, B. A. , Shah, A. A. , Gray, M. M. , Casner, K. L. , Barthelet, A. , Messer, P. W. , Simmons, M. P. , Guayasamin, J. M. , Encalada, A. C. , Kondratieff, B. C. , Flecker, A. S. , Thomas, S. A. , Ghalambor, C. K. , Poff, N. L. , Funk, W. C. , & Zamudio, K. R. (2018). Narrow thermal tolerance and low dispersal drive higher speciation in tropical mountains. Proceedings of the National Academy of Sciences of the United States of America, 115, 12471–12476. 10.1073/pnas.1809326115 30397141 PMC6298121

[ece39349-bib-0096] Porter, W. P. , Sabo, J. L. , Tracy, C. R. , Reichman, O. J. , & Ramankutty, N. (2002). Physiology on a landscape scale: Plant‐animal interactions. Integrative and Comparative Biology, 42, 431–453. 10.1093/icb/42.3.431 21708738

[ece39349-bib-0097] Potter, K. A. , Woods, H. A. , & Pincebourde, S. (2013). Microclimatic challenges in global change biology. Global Change Biology, 19, 2932–2939. 10.1111/gcb.12257 23681970

[ece39349-bib-0098] Pujol, B. , Wilson, A. J. , Ross, R. I. C. , & Pannell, J. R. (2008). Are Q(ST)‐F(ST) comparisons for natural populations meaningful? Molecular Ecology, 17, 4782–4785. 10.1111/j.1365-294X.2008.03958.x 19140971

[ece39349-bib-0099] Qu, Y.‐F. , & Wiens, J. J. (2020). Higher temperatures lower rates of physiological and niche evolution. Proceedings of the Royal Society, 287(20200823). 10.1098/rspb.2020.0823 PMC7423657 32673554

[ece39349-bib-0100] R Core Team . (2019). R: A Language and Environment for Statistical Computing.

[ece39349-bib-0101] Ragland, G. J. , & Kingsolver, J. G. (2008). Evolution of thermotolerance in seasonal environments: The effects of annual temperature variation and life‐history timing in *Wyeomyia smithii* . Evolution, 62, 1345–1357. 10.1111/j.1558-5646.2008.00367.x 18331458

[ece39349-bib-0102] Raymond, M. , & Rousset, F. (1995). GENEPOP (version 1.2): Population genetics software for exact tests and ecumenicism. The Journal of Heredity, 86, 248–249. 10.1093/oxfordjournals.jhered.a111573

[ece39349-bib-0103] Remold, S. (2012). Understanding specialism when the Jack of all trades can be the master of all. Proceedings of the Biological Sciences, 279, 4861–4869. 10.1098/rspb.2012.1990 PMC3497242 23097515

[ece39349-bib-0104] Richter‐Boix, A. , Katzenberger, M. , Duarte, H. , Quintela, M. , Tejedo, M. , & Laurila, A. (2015). Local divergence of thermal reaction norms among amphibian populations is affected by pond temperature variation. Evolution, 69, 2210–2226. 10.1111/evo.12711 26118477

[ece39349-bib-0105] Ruthsatz, K. , Dausmann, K. H. , Peck, M. A. , & Glos, J. (2022). Thermal tolerance and acclimation capacity in the European common frog (*Rana temporaria*) change throughout ontogeny. Journal of Experimental Zoology Part A: Ecological Genetics and Physiology, 337, 477–490. 10.1002/jez.2582 35226414

[ece39349-bib-0106] Saint‐Pé, K. , Leitwein, M. , Tissot, L. , Poulet, N. , Guinand, B. , Berrebi, P. , Marselli, G. , Lascaux, J.‐M. , Gagnaire, P.‐A. , & Blanchet, S. (2019). Development of a large SNPs resource and a low‐density SNP array for brown trout (*Salmo trutta*) population genetics. BMC Genomics, 20, 582. 10.1186/s12864-019-5958-9 31307373 PMC6631668

[ece39349-bib-0107] Schou, M. F. , Mouridsen, M. B. , Sørensen, J. G. , & Loeschcke, V. (2017). Linear reaction norms of thermal limits in Drosophila: Predictable plasticity in cold but not in heat tolerance. Functional Ecology, 31, 934–945. 10.1111/1365-2435.12782

[ece39349-bib-0108] Senior, A. F. , Atkins, Z. S. , Clemann, N. , Gardner, M. G. , Schroder, M. , While, G. M. , Wong, B. B. M. , & Chapple, D. G. (2019). Variation in thermal biology of three closely related lizard species along an elevation gradient. Biological Journal of the Linnean Society, 127, 278–291. 10.1093/biolinnean/blz046

[ece39349-bib-0109] Seppä, P. , & Laurila, A. (1999). Genetic structure of Island populations of the anurans *Rana temporaria* and *Bufo bufo* . Heredity, 82, 309–317. 10.1038/sj.hdy.6884900 10336706

[ece39349-bib-0110] Shah, A. A. , Gill, B. A. , Encalada, A. C. , Flecker, A. S. , Funk, W. C. , Guayasamin, J. M. , Kondratieff, B. C. , Poff, N. L. R. , Thomas, S. A. , Zamudio, K. R. , & Ghalambor, C. K. (2017). Climate variability predicts thermal limits of aquatic insects across elevation and latitude. Functional Ecology, 31, 2118–2127. 10.1111/1365-2435.12906

[ece39349-bib-0111] Slatkin, M. (1973). Gene flow and selection in a cline. Genetics, 75, 733–356. 10.1093/genetics/75.4.733 4778791 PMC1213045

[ece39349-bib-0112] Slatyer, R. A. , Nash, M. A. , & Hoffmann, A. A. (2016). Scale‐dependent thermal tolerance variation in Australian mountain grasshoppers. Ecography, 39, 572–582. 10.1111/ecog.01616

[ece39349-bib-0113] Slatyer, R. A. , & Schoville, S. D. (2016). Physiological limits along an elevational gradient in a radiation of montane ground beetles. PLoS One, 11, e0151959. 10.1371/journal.pone.0151959 27043311 PMC4820226

[ece39349-bib-0114] Slatyer, R. A. , Schoville, S. D. , Nufio, C. R. , & Buckley, L. B. (2019). Do different rates of gene flow underlie variation in phenotypic and phenological clines in a montane grasshopper community? Ecology and Evolution, 10, 980–997. 10.1002/ece3.5961 32015859 PMC6988534

[ece39349-bib-0115] Socolar, J. B. , Epanchin, P. N. , Beissinger, S. R. , & Tingley, M. W. (2017). Phenological shifts conserve thermal niches in North American birds and reshape expectations for climate‐driven range shifts. Proceedings of the National Academy of Sciences of the United States of America, 114, 12976–12981. 10.1073/pnas.1705897114 29133415 PMC5724251

[ece39349-bib-0116] Somero, G. N. (2010). The physiology of climate change: How potentials for acclimatization and genetic adaptation will determine ‘winners’ and ‘losers.’. The Journal of Experimental Biology, 213, 912–920. 10.1242/jeb.037473 20190116

[ece39349-bib-0117] Sørensen, J. G. , Norry, F. M. , Scannapieco, A. C. , & Loeschcke, V. (2005). Altitudinal variation for stress resistance traits and thermal adaptation in adult *Drosophila buzzatii* from the New World. Journal of Evolutionary Biology, 18, 829–837. 10.1111/j.1420-9101.2004.00876.x 16033554

[ece39349-bib-0118] Stevens, G. C. (1989). The latitudinal gradient in geographical range—how so many species coexist in the tropics. The American Naturalist, 133, 240–256. 10.1086/284913

[ece39349-bib-0119] Stillman, J. H. (2003). Acclimation capacity underlies susceptibility to climate change. Science, 301, 65. 10.1126/science.1083073 12843385

[ece39349-bib-0120] Suggitt, A. J. , Gillingham, P. K. , Hill, J. K. , Huntley, B. , Kunin, W. E. , Roy, D. B. , & Thomas, C. D. (2011). Habitat microclimates drive fine‐scale variation in extreme temperatures. Oikos, 120, 1–8. 10.1111/j.1600-0706.2010.18270.x

[ece39349-bib-0121] Sunday, J. , Bennett, J. M. , Calosi, P. , Clusella‐Trullas, S. , Gravel, S. , Hargreaves, A. L. , Leiva, F. P. , Verberk, W. C. E. P. , Olalla‐Tárraga, M. Á. , & Morales‐Castilla, I. (2019). Thermal tolerance patterns across latitude and elevation. Philosophical Transactions of the Royal Society B: Biological Sciences, 374, 20190036. 10.1098/rstb.2019.0036 PMC6606462 31203755

[ece39349-bib-0122] Sunday, J. M. , Bates, A. E. , Kearney, M. R. , Colwell, R. K. , Dulvy, N. K. , Longino, J. T. , & Huey, R. B. (2014). Thermal‐safety margins and the necessity of thermoregulatory behavior across latitude and elevation. Proceedings of the National Academy of Sciences of the United States of America, 111, 5610–5615. 10.1073/pnas.1316145111 24616528 PMC3992687

[ece39349-bib-0123] Tonione, M. A. , Cho, S. M. , Richmond, G. , Irian, C. , & Tsutsui, N. D. (2020). Intraspecific variation in thermal acclimation and tolerance between populations of the Winter ant, Prenolepis imparis. Ecology and Evolution, 10, 749–4761. 10.1002/ece3.6229 PMC7297759 32551058

[ece39349-bib-0124] Walther, G.‐R. , Post, E. , Convey, P. , Menzel, A. , Parmesan, C. , Beebee, T. J. C. , Fromentin, J. M. , Hoegh‐Guldberg, O. , & Bairlein, F. (2002). Ecological responses to recent climate change. Nature, 416, 389–395. 10.1038/416389a 11919621

[ece39349-bib-0125] Weir, B. S. , & Cockerham, C. C. (1984). Estimating F‐statistics for the analysis of population structure. Evolution, 38, 1358–1370. 10.2307/2408641 28563791

[ece39349-bib-0126] Wilczek, A. M. , Burghardt, L. T. , Cobb, A. R. , Cooper, M. D. , Welch, S. M. , & Schmitt, J. (2010). Genetic and physiological bases for phenological responses to current and predicted climates. Philosophical Transactions of the Royal Society B, 365, 3129–3147. 10.1098/rstb.2010.0128 PMC2981944 20819808

