# Supplementary material for: Phenology and plasticity can prevent adaptive clines in thermal tolerance across temperate mountains: The importance of the elevation‐time axis
Source: Ecol Evol. 2022 Oct 5;12(10):e9349. doi: 10.1002/ece3.9349 (PMC9534760; doi:10.1002/ece3.9349)
Supplement: Supplementary file 1 — Appendix S1 [file ECE3-12-e9349-s001.docx]

SUPPORTING INFORMATION

Additional supporting information may be found in the online version of this article.

Text 1

Four microsatellites (Rtempμ1, Rtempμ2, Rtempμ4, RtμB) were amplified by using 6-20 ng of template DNA, 0.3-0.7 μM of primer, 250 μM of dNTPs (PROMEGA, USA) and Go Taq ® Flexi DNA Polymerase of Promega, USA: 0.5U of Go Taq Polymerase, 2.0-2.5 mM of Mg2+, 2 μl of 5x colorless Go Taq Flexi Buffer and 2 μl of 5x Green Go Taq Flexi Buffer. PCR cycles were starting with 5 min at 94 °C and 40 cycles that consisted of: denaturation, 30 s at 94 °C; annealing, 30 s at 46 or 58 °C (depending on primer); and extending, 30 s at 72 °C. After 40 cycles, twenty minutes at 72 °C were left for elongation. The remainder seven primers were processed with multiplex PCR; we used 6-20 ng of template DNA, 0.3-0.7 μM of primer (RtμH, RtU4, and RtU7 for A-plex, and BFG072, BFG093, BFG183, and BFG241 for B-plex PCR), 5 μl of Qiagen Multiplex PCR Master Mix (Quiagen GmbH, Hilden, Germany). PCR cycles were starting with 15 min at 95 °C and 38 cycles that consisted of: denaturation, 30 s at 94 °C; annealing, 30 s at 55 °C; and extending, 30 s at 72 °C; and finally elongation during 30 min at 60 °C. PCR reactions were performed on Applied Biosystems 2720 Thermal (Applied Biosystems, Inc.), GeneAmp®PCR Systems 9700 (Applied Biosystems, Inc.) and Bio-Rad My Cycler™ (Bio-Rad Laboratories, Inc). PCR products were segregated and detected by capillary electrophoresis on an ABI PRISM® 3130xl Genetic Analyzer (Applied Biosystems).

|  | PUR | NUE | COL | VIA | COR | FAN | PAC | PAN | ALI | SEN | LLA |
| --- | --- | --- | --- | --- | --- | --- | --- | --- | --- | --- | --- |
| PUR^¥^ | - | **0.041** | **0.011** | **<0.001** | 0.198 | 0.997 | **<0.001** | 0.375 | **<0.001** | 0.366 | **<0.001** |
| NUE | **<0.001** | - | 1 | 0.838 | 0.999 | 0.375 | 0.917 | 0.997 | **<0.001** | 0.999 | 0.950 |
| COL | **<0.001** | 0.135 | - | 0.973 | 0.994 | 0.160 | 0.992 | 0.956 | **<0.001** | 0.970 | 0.996 |
| VIA | **<0.001** | 0.767 | 0.991 | - | 0.436 | **<0.001** | 1 | 0.243 | 0.144 | 0.295 | 1 |
| COR | **<0.010** | 0.667 | **<0.001** | **<0.010** | - | 0.787 | 0.569 | 1 | **<0.001** | 1 | **0.013** |
| FAN | 0.064 | **0.023** | **<0.001** | **<0.001** | 0.954 | - | **<0.010** | 0.931 | **<0.001** | 0.922 | 0.013 |
| PAC | **<0.001** | 0.999 | **0.011** | 0.250 | 0.945 | 0.103 | - | 0.347 | 0.091 | 0.408 | 1 |
| PAN | **<0.001** | 1 | 0.115 | 0.765 | 0.538 | **<0.010** | 0.999 | - | **<0.001** | 1 | 0.442 |
| ALI | **<0.001** | 1 | 0.115 | 0.765 | 0.539 | **<0.010** | 0.999 | 1 | - | **<0.001** | 0.100 |
| SEN | **<0.001** | 1 | 0.056 | 0.588 | 0.710 | **0.022** | 1 | 1 | 1 | - | 0.504 |
| LLA | **<0.001** | 1 | 0.217 | 0.883 | 0.469 | **<0.010** | 0.997 | 1 | 1 | 1 | - |
|  |  |  |  |  |  |  |  |  |  |  |  |

**Table S1**. Pairwise comparison (Tukey HSD p-values) of critical thermal maximum (CT_max_) and minimum (CT_min_) between 11 populations of R. parvipalmata. CT_max_ comparisons in upper diagonal and CT_min_ comparisons in lower diagonal. Significant differences are marked in bold.^¥^ PUR, Purón; NUE, Nueva; COL, Color; VIA, Viango; COR, Cortegueros; FAN, Fana; PAC, Pandecarmen; PAN, Pandébano; ALI, Aliva; SEN, Señales, LLA, Llagusecu.

**Table S2.** Monthly average minimum temperatures, mean and the lowest minimum temperature (TMIN) (WorldClim database, Hijmans 2014), for the eleven studied populations of *R. parvipalmata*, with their longitude and latitude (in decimal degrees, WGS84) and elevation (m a.s.l.). The shaded values correspond to the period of presence of larvae in ponds. Mean and TMIN values have been calculated only for the period within shaded months.

| Population | Elevation | Longitude | Latitude | JAN | FEB | MAR | APR | MAY | JUN | JUL | AGO | SEP | OCT | NOV | DIC | $\bar{\mathrm{Mean}}$ | TMIN |
| --- | --- | --- | --- | --- | --- | --- | --- | --- | --- | --- | --- | --- | --- | --- | --- | --- | --- |
| PURON | 40 | -4.698653 | 43.3794037 | 4.7 | 5.8 | 7.1 | 8 | 10.3 | 12.9 | 14.8 | 15.3 | 14.2 | 11.3 | 7.8 | 6 | 8.11 | 4.70 |
| NUEVA | 140 | -4.933516 | 43.4251387 | 5 | 5.7 | 6.9 | 7.7 | 10.1 | 12.9 | 14.6 | 15.1 | 13.9 | 10.9 | 7.7 | 6 | 7.97 | 5.00 |
| COLOR | 380 | -5.276755 | 43.4251387 | 3.0 | 3.3 | 5 | 5.9 | 8.5 | 11.5 | 13.7 | 14.3 | 12.6 | 9.2 | 5.7 | 4.4 | 6.40 | 3.00 |
| VIANGO | 480 | -4.809492 | 43.3710238 | 1.9 | 2.8 | 4.7 | 5.7 | 8.5 | 11.3 | 13.7 | 14.4 | 12.6 | 9.4 | 5.4 | 3.6 | 5.76 | 1.90 |
| CORTEGUEROS | 650 | -4.939555 | 43.3174303 | 1.8 | 2.6 | 4.6 | 5.5 | 8.3 | 11.2 | 13.7 | 14.2 | 12.4 | 9.1 | 5.1 | 3.4 | 5.31 | 1.80 |
| FANA | 950 | -5.0131 | 43.279605 | -0.2 | 0.2 | 2.1 | 3.4 | 6.7 | 9.8 | 12.8 | 13.4 | 11.2 | 7.4 | 3.5 | 1.6 | 3.67 | -0.20 |
| PANDECARMEN | 1100 | -5.014256 | 43.263259 | -0.7 | -0.3 | 1.5 | 2.8 | 6.3 | 9.4 | 12.5 | 13.2 | 10.9 | 6.9 | 3 | 1.1 | 3.94 | -0.30 |
| PANDEBANO | 1200 | -4.786842 | 43.232574 | -0.9 | -0.8 | 0.4 | 1.4 | 5.2 | 8.5 | 11.7 | 12.4 | 10.1 | 6.1 | 2.6 | 0.7 | 2.94 | -0.80 |
| ALIVA | 1400 | -4.764802 | 43.1788459 | -1.1 | -1.1 | -0.5 | 0.2 | 4 | 7.6 | 11.2 | 11.9 | 9.3 | 5.4 | 2.1 | 0.7 | 4.50 | -0.50 |
| SEÑALES | 1600 | -5.246499 | 43.0791979 | -3.2 | -3.3 | -2.2 | -1.2 | 2.6 | 6.4 | 10.7 | 11.3 | 8.5 | 4.1 | 0.7 | -0.9 | 4.62 | -1.20 |
| LLAGUSECU | 1800 | -4.992107 | 43.22258 | -3.9 | -4.6 | -3.6 | -2.6 | 1.4 | 5 | 9.6 | 10.3 | 7.5 | 3.1 | -0.1 | -1.3 | 6.58 | 1.40 |

**Table S3.**  Monthly average maximum temperatures, mean and the highest maximum temperature (TMAX) (WorldClim database Hijmans 2014) for the eleven studied populations of *R. parvipalmata,* with their longitude and latitude (in decimal degrees, WGS84) and elevation (m a.s.l.). The shaded values correspond to the period of presence of larvae in the ponds. Mean and TMAX values have been calculated only for the period within shaded months.

| Population | Elevation | Longitude | Latitude | JAN | FEB | MAR | APR | MAY | JUN | JUL | AGO | SEP | OCT | NOV | DIC | Mean | TMAX |
| --- | --- | --- | --- | --- | --- | --- | --- | --- | --- | --- | --- | --- | --- | --- | --- | --- | --- |
| PURON | 40 | -4.698653 | 43.3794037 | 12.3 | 13.3 | 14.6 | 15.5 | 17.8 | 20.5 | 22.3 | 22.9 | 21.7 | 18.9 | 15.4 | 13.6 | 15.66 | 21.7 |
| NUEVA | 140 | -4.933516 | 43.4251387 | 11.9 | 12.7 | 13.9 | 14.6 | 17 | 19.8 | 21.8 | 22.4 | 21.1 | 18.2 | 14.9 | 13.3 | 15.08 | 21.1 |
| COLOR | 380 | -5.276755 | 43.4251387 | 9.9 | 10.1 | 11.9 | 12.8 | 15.4 | 18.4 | 20.3 | 20.8 | 19.1 | 15.8 | 12.3 | 10.9 | 13.13 | 19.1 |
| VIANGO | 480 | -4.809492 | 43.3710238 | 9.3 | 10.1 | 12 | 13 | 15.9 | 18.7 | 20.4 | 21.1 | 19.3 | 16.1 | 12.1 | 10.3 | 12.78 | 19.3 |
| CORTEGUEROS | 650 | -4.939555 | 43.3174303 | 9.4 | 10.2 | 12.2 | 13.2 | 15.9 | 18.9 | 20.6 | 21.2 | 19.4 | 16.1 | 12.1 | 10.4 | 12.79 | 18.9 |
| FANA | 950 | -5.0131 | 43.279605 | 7.2 | 7.6 | 9.5 | 10.8 | 14.1 | 17.2 | 18.8 | 19.5 | 17.3 | 13.4 | 9.5 | 7.6 | 11.07 | 17.2 |
| PANDECARMEN | 1100 | -5.014256 | 43.263259 | 6.7 | 7.1 | 8.8 | 10.2 | 13.6 | 16.8 | 18.5 | 19.1 | 16.8 | 12.9 | 9 | 7 | 11.30 | 16.8 |
| PANDEBANO | 1200 | -4.786842 | 43.232574 | 6 | 6.1 | 7.3 | 8.3 | 12.1 | 15.4 | 17.4 | 18.1 | 15.7 | 11.8 | 8.2 | 6.4 | 9.84 | 15.4 |
| ALIVA | 1400 | -4.764802 | 43.1788459 | 5.5 | 5.5 | 6.1 | 6.8 | 10.7 | 14.2 | 16.3 | 17 | 14.5 | 10.6 | 7.3 | 5.9 | 10.82 | 16.3 |
| SEÑALES | 1600 | -5.246499 | 43.0791979 | 4.4 | 4.4 | 5.4 | 6.5 | 10.3 | 14 | 15.6 | 16.3 | 13.5 | 9 | 5.7 | 4.1 | 11.60 | 15.6 |
| LLAGUSECU | 1800 | -4.992107 | 43.22258 | 3.1 | 2.3 | 3.3 | 4.3 | 8.3 | 11.9 | 13.4 | 14.1 | 11.2 | 6.9 | 3.6 | 2.4 | 11.92 | 14.1 |

**Table S4.** Monthly temperature range, calculated as TMAX-TMIN for each month and Seasonal range (SR), calculated as the mean monthly temperature range for the period when larvae are present in the ponds (shaded values), for the eleven studied populations of *R. parvipalmata,* with their longitude and latitude (in decimal degrees, WGS84) and elevation (m a.s.l.).

| Population | Elevation | longitude | latitude | JAN | FEB | MAR | APR | MAY | JUN | JUL | AGO | SEP | OCT | NOV | DIC | SR |
| --- | --- | --- | --- | --- | --- | --- | --- | --- | --- | --- | --- | --- | --- | --- | --- | --- |
| PURON | 40 | -4.698653 | 43.3794037 | 7.60 | 7.50 | 7.50 | 7.50 | 7.50 | 7.60 | 7.50 | 7.60 | 7.50 | 7.60 | 7.60 | 7.60 | 7.55 |
| NUEVA | 140 | -4.933516 | 43.4251387 | 6.90 | 7.00 | 7.00 | 6.90 | 6.90 | 6.90 | 7.20 | 7.30 | 7.20 | 7.30 | 7.20 | 7.30 | 7.10 |
| COLOR | 380 | -5.276755 | 43.4251387 | 6.90 | 6.80 | 6.90 | 6.90 | 6.90 | 6.90 | 6.60 | 6.50 | 6.50 | 6.60 | 6.60 | 6.50 | 6.73 |
| VIANGO | 480 | -4.809492 | 43.3710238 | 7.40 | 7.30 | 7.30 | 7.30 | 7.40 | 7.40 | 6.70 | 6.70 | 6.70 | 6.70 | 6.70 | 6.70 | 7.01 |
| CORTEGUEROS | 650 | -4.939555 | 43.3174303 | 7.60 | 7.60 | 7.60 | 7.70 | 7.60 | 7.70 | 6.90 | 7.00 | 7.00 | 7.00 | 7.00 | 7.00 | 7.47 |
| FANA | 950 | -5.0131 | 43.279605 | 7.40 | 7.40 | 7.40 | 7.40 | 7.40 | 7.40 | 6.00 | 6.10 | 6.10 | 6.00 | 6.00 | 6.00 | 7.40 |
| PANDECARMEN | 1100 | -5.014256 | 43.263259 | 7.40 | 7.40 | 7.30 | 7.40 | 7.30 | 7.40 | 6.00 | 5.90 | 5.90 | 6.00 | 6.00 | 5.90 | 7.36 |
| PANDEBANO | 1200 | -4.786842 | 43.232574 | 6.90 | 6.90 | 6.90 | 6.90 | 6.90 | 6.90 | 5.70 | 5.70 | 5.60 | 5.70 | 5.60 | 5.70 | 6.90 |
| ALIVA | 1400 | -4.764802 | 43.1788459 | 6.60 | 6.60 | 6.60 | 6.60 | 6.70 | 6.60 | 5.10 | 5.10 | 5.20 | 5.20 | 5.20 | 5.20 | 6.32 |
| SEÑALES | 1600 | -5.246499 | 43.0791979 | 7.60 | 7.70 | 7.60 | 7.70 | 7.70 | 7.60 | 4.90 | 5.00 | 5.00 | 4.90 | 5.00 | 5.00 | 6.98 |
| LLAGUSECU | 1800 | -4.992107 | 43.22258 | 7.00 | 6.90 | 6.90 | 6.90 | 6.90 | 6.90 | 3.80 | 3.80 | 3.70 | 3.80 | 3.70 | 3.70 | 5.35 |

**Table S5.** Microenvironmental pond temperature for the eleven studied populations of *R. parvipalmata* (Pop). Elev: Elevation*.* N: number of days of temperature monitoring. Start and End date: period of pond temperature monitoring. ABS: absolute, mean ± SE: mean ± standard error, of daily maximum (tmax) and minimum (tmin) temperatures. Date Abs: the exact day when these extreme temperatures were recorded.

| tmin | | | | | | | | | | | tmax | | | |  |  |  |  |  |  |  |  |  |  |
| --- | --- | --- | --- | --- | --- | --- | --- | --- | --- | --- | --- | --- | --- | --- | --- | --- | --- | --- | --- | --- | --- | --- | --- | --- |
| Pop | | Elev N | | | Start date | | | End date | ABS | | $\bar{X}$± SE | Date Abs | | | | ABS | | $\bar{X}$± SE | | Date Abs | | | |  |
| Purón | | 40 | 101 | | 08/02/2013 | 23/05/2013 | | 9.9 | 10.3 ± 0.1 | | | 24/02/2013 | | 11.4 | | 10.7 ± 0.1 | | | | | 28/04/2013 | | |  |
| Nueva | | 140 | 80 | | 06/02/2013 | 26/04/2013 | | 6.4 | 9.3 ± 0.1 | | | 23/02/2013 | | 15.7 | | 11.0 ± 0.2 | | | | | 25/04/2013 | | |  |
| Color | | 380 | 506 | | 01/09/2008 | 31/05/2010 | | 3.8 | 9.9 ± 0.1 | | | 08/01/2010 | | 22.5 | | 11.4 ± 0.1 | | | | | 30/05/2010 | | |  |
| Viango | | 480 | 81 | | 09/02/2013 | 30/04/2013 | | 2.4 | 7.4 ± 0.3 | | | 11/02/2013 | | 25.6 | | 12.3 ± 0.5 | | | | | 25/04/2013 | | |  |
| Cortegueros | | 650 | 140 | | 09/02/2013 | 28/06/2013 | | 1.0 | 7.6 ± 0.5 | | | 27/02/2013 | | 28.4 | | 13.9 ± 0.5 | | | | | 12/06/2013 | | |  |
| Fana | | 950 | 42 | | 26/02/2014 | 08/04/2014 | | 1.5 | 4.4 ± 0.3 | | | 23/03/2014 | | 29.3 | | 13.3 ± 1.0 | | | | | 06/04/2014 | | |  |
| Pandecarmen | | 1100 | 40 | | 07/03/2014 | 15/05/2014 | | 0.2 | 2.7 ± 0.5 | | | 15/03/2014 | | 33.0 | | 15.8 ± 1.5 | | | | | 18/04/2014 | | |  |
| Pandébano | | 1200 | 113 | | 10/03/2014 | 30/06/2014 | | 0.6 | 7.8 ± 0.3 | | | 30/03/2014 | | 28.9 | | 15.6 ± 0.6 | | | | | 13/06/2014 | | |  |
| Aliva | | 1400 | 82 | | 29/04/2002 | 18/07/2002 | | 4.3 | 10.5 ± 0.4 | | | 09/05/2002 | | 24.5 | | 17.0 ± 0.5 | | | | | 17/06/2002 | | |  |
| Señales | | 1600 | 83 | | 02/05/2012 | 23/04/2013 | | 4.0 | 9.0 ± 0.4 | | | 03/05/2012 | | 22.6 | | 14.0 ± 0.4 | | | | | 18/07/2012 | | |  |
| Llagusecu | | 1800 | 114 | | 11/08/2009 | 01/08/2010 | | 0.5 | 6.8 ± 0.5 | | | 06/05/2010 | 29.7 | | | | | 12.4 ± 0.9 | | 19/08/2009 | | | | |

**Table S6.** Mean values temperature ± SD (°C) for the constant acclimation treatments.

| Acclimation Treatment | $\bar{\boldsymbol{X}}$± SD (°C) |
| --- | --- |
| 6 | 5.92 ± 0.32 |
| 13 | 12.75 ± 0.18 |
| 20 | 19.96 ± 0.26 |
| 27 | 26.83 ± 0.19 |

**Table S7.** Molecular markers used in this study and the PCR conditions.

|  |  |  |  |  | **PCR conditions** | | |  |
| --- | --- | --- | --- | --- | --- | --- | --- | --- |
| **Locus** | **Repeat motif** | **Allele size range (bp)** | **Number of alleles** | **Dye** | **Polymerase** | **T^a^ (^o^C)** | **cycles** | **Gene Bank no.** |
| Rtempμ1 | (CA)_4_GG(CA)_24_ | 92-130 | 21 | PET | GoTaq | 46 | 40 | AF297972 |
| Rtempμ2 | (AT)_8_AT(AC)_22_ | 83-140 | 7 | PET | GoTaq | 46 | 40 | AF297973 |
| Rtempμ4 | (AC)_16_ | 106-142 | 15 | VIC | GoTaq | 58 | 40 | AF297975 |
| RtμB | (CA)_14_ | 246 | 27 | VIC | GoTaq | 58 | 40 | AF489577 |
| *A-plex* |  |  |  |  | Qiagen | 55 | 40 |  |
| RtμH | (CA)_7_ | 206 | 4 | NED |  |  |  | AF489579 |
| RtU4 | (GT)_23_(T)_13_ | 75-108 | 14 | VIC |  |  |  | AF257481 |
| RtU7 | (GATA)_37_ | 152-295 | 57 | FAM |  |  |  | AF257482 |
| *B-plex* |  |  |  |  | Qiagen | 55 | 38 |  |
| BFG072 | (TGTA)_13_ | 104-132 | 2 | PET |  |  |  | EU334947 |
| BFG093 | (TG)_21_ | 116-142 | 20 | FAM |  |  |  | EU334958 |
| BFG183 | (TG)_9_ | 112-158 | 41 | NED |  |  |  | EU335004 |
| BFG241 | (CATA)_8_ | 107-152 | 21 | VIC |  |  |  | EU335033 |

**Table S8** Pairwise F_ST_ values for the eleven populations included in the study.

|  | NUE | COL | VIA | COR | FAN | PAC | PAN | ALI | SEN | LLA |
| --- | --- | --- | --- | --- | --- | --- | --- | --- | --- | --- |
| PUR^¥^ | 0.0437 | 0.0670 | 0.0201 | 0.0542 | 0.0465 | 0.0363 | 0.0851 | 0.0578 | 0.0817 | 0.0701 |
| NUE |  | 0.0365 | 0.0393 | 0.0253 | 0.0178 | 0.0125 | 0.1024 | 0.1081 | 0.0622 | 0.0708 |
| COL |  |  | 0.0603 | 0.0536 | 0.0378 | 0.0501 | 0.1002 | 0.0936 | 0.0642 | 0.1175 |
| VIA |  |  |  | 0.0627 | 0.0499 | 0.0247 | 0.0714 | 0.0601 | 0.0907 | 0.0560 |
| COR |  |  |  |  | 0.0143 | 0.0334 | 0.1152 | 0.1165 | 0.0350 | 0.0934 |
| FAN |  |  |  |  |  | 0.0164 | 0.0802 | 0.0958 | 0.0389 | 0.0777 |
| PAC |  |  |  |  |  |  | 0.0781 | 0.1006 | 0.0627 | 0.0330 |
| PAN |  |  |  |  |  |  |  | 0.0700 | 0.1115 | 0.0928 |
| ALI |  |  |  |  |  |  |  |  | 0.0896 | 0.1322 |
| SEN |  |  |  |  |  |  |  |  |  | 0.1293 |

PUR, Purón; NUE, Nueva; COL, Color; VIA, Viango; COR, Cortegueros; FAN, Fana; PAC, Pandecarmen; PAN, Pandébano; ALI, Aliva; SEN, Señales, LLA, Llagusecu.

**Table S9.** Pairwise P_ST_ values for CT_max_ (upper diagonal) and CT*_min_* (lower diagonal), under the null assumption c=h^2^=1.

|  | PUR | NUE | COL | VIA | COR | FAN | PAC | PAN | ALI | SEN | LLA |
| --- | --- | --- | --- | --- | --- | --- | --- | --- | --- | --- | --- |
| PUR^¥^ | - | 0.3468 | 0.3451 | 0.4301 | 0.2286 | 0.0226 | 0.4293 | 0.1774 | 0.6887 | 0.1596 | 0.4204 |
| NUE | 0.6171 | - | 0.0000 | 0.0495 | 0.0000 | 0.2192 | 0.0378 | 0.0035 | 0.4018 | 0.0000 | 0.0294 |
| COL | 0.7715 | 0.3939 | . | 0.0091 | 0.0000 | 0.2314 | 0.0000 | 0.0279 | 0.3130 | 0.0162 | 0.0000 |
| VIA | 0.5746 | 0.0485 | 0.0000 | - | 0.1001 | 0.3386 | 0.0000 | 0.1319 | 0.1427 | 0.1112 | 0.0000 |
| COR | 0.3566 | 0.1759 | 0.5650 | 0.2282 | - | 0.1060 | 0.0882 | 0.0000 | 0.4403 | 0.0000 | 0.0782 |
| FAN | 0.2590 | 0.5517 | 0.7791 | 0.4360 | 0.0778 | - | 0.3344 | 0.0617 | 0.6445 | 0.0539 | 0.3250 |
| PAC | 0.4345 | 0.0000 | 0.2776 | 0.0759 | 0.0177 | 0.2023 | - | 0.1203 | 0.1707 | 0.0999 | 0.0000 |
| PAN | 0.6232 | 0.0000 | 0.3513 | 0.0466 | 0.1827 | 0.5241 | 0.0000 | - | 0.4667 | 0.0000 | 0.1096 |
| ALI | 0.6419 | 0.0000 | 0.3894 | 0.0507 | 0.2060 | 0.5717 | 0.0000 | 0.0000 | - | 0.4295 | 0.1720 |
| SEN | 0.4391 | 0.0000 | 0.1789 | 0.0312 | 0.0441 | 0.2233 | 0.0000 | 0.0000 | 0.0000 | - | 0.0892 |
| LLA | 0.6057 | 0.0000 | 0.2828 | 0.0242 | 0.1786 | 0.4995 | 0.0000 | 0.0000 | 0.0000 | 0.0000 | - |

^¥^ PUR, Purón; NUE, Nueva; COL, Color; VIA, Viango; COR, Cortegueros; FAN, Fana; PAC, Pandecarmen; PAN, Pandébano; ALI, Aliva; SEN, Señales, LLA, Llagusecu.

**Table S10.** Confidence interval in global P_ST_ for CT_max_ and CT_min_ estimates with different values of c and h^2^ using non-parametric bootstrap.

| CTmax | | | | | | | | | | | | |
| --- | --- | --- | --- | --- | --- | --- | --- | --- | --- | --- | --- | --- |
| c | h=1 | | | h=0.75 | | | h=0.5 | | | h=0.25 | | |
|  | **Low** | **Mean** | **Up** | **Low** | **Mean** | **Up** | **Low** | **Mean** | **Up** | **Low** | **Mean** | **Up** |
| 1 | 0.12 | 0.17 | 0.21 | 0.15 | 0.20 | 0.25 | 0.19 | 0.25 | 0.31 | 0.27 | 0.35 | 0.42 |
| 0.9 | 0.11 | 0.16 | 0.20 | 0.14 | 0.19 | 0.24 | 0.18 | 0.24 | 0.30 | 0.26 | 0.33 | 0.41 |
| 0.8 | 0.10 | 0.14 | 0.19 | 0.13 | 0.17 | 0.22 | 0.17 | 0.22 | 0.28 | 0.24 | 0.32 | 0.39 |
| 0.7 | 0.09 | 0.13 | 0.17 | 0.12 | 0.16 | 0.21 | 0.15 | 0.21 | 0.26 | 0.23 | 0.30 | 0.37 |
| 0.6 | 0.08 | 0.12 | 0.15 | 0.10 | 0.15 | 0.19 | 0.14 | 0.19 | 0.24 | 0.21 | 0.27 | 0.34 |
| 0.5 | 0.07 | 0.10 | 0.14 | 0.09 | 0.13 | 0.17 | 0.12 | 0.17 | 0.22 | 0.19 | 0.25 | 0.32 |
| 0.4 | 0.06 | 0.09 | 0.12 | 0.08 | 0.11 | 0.14 | 0.10 | 0.14 | 0.19 | 0.17 | 0.22 | 0.28 |
| 0.3 | 0.05 | 0.07 | 0.09 | 0.06 | 0.09 | 0.12 | 0.08 | 0.12 | 0.16 | 0.14 | 0.19 | 0.24 |
| 0.2 | 0.03 | 0.05 | 0.07 | 0.04 | 0.06 | 0.08 | 0.06 | 0.09 | 0.12 | 0.10 | 0.15 | 0.19 |
| 0.1 | 0.02 | 0.03 | 0.04 | 0.02 | 0.03 | 0.05 | 0.03 | 0.05 | 0.07 | 0.06 | 0.09 | 0.12 |
| CTmin | | | | | | | | | | | | |
| c |  | h=1 |  |  | h=0.75 |  |  | h=0.5 |  |  | h=0.25 |  |
|  | **Low** | **Mean** | **Up** | **Low** | **Mean** | **Up** | **Low** | **Mean** | **Up** | **Low** | **Mean** | **Up** |
| 1 | 0.17 | 0.24 | 0.30 | 0.20 | 0.27 | 0.34 | 0.25 | 0.32 | 0.40 | 0.32 | 0.41 | 0.50 |
| 0.9 | 0.16 | 0.22 | 0.28 | 0.19 | 0.26 | 0.33 | 0.23 | 0.31 | 0.39 | 0.31 | 0.40 | 0.49 |
| 0.8 | 0.15 | 0.21 | 0.27 | 0.18 | 0.24 | 0.31 | 0.22 | 0.29 | 0.37 | 0.29 | 0.38 | 0.47 |
| 0.7 | 0.14 | 0.19 | 0.25 | 0.17 | 0.23 | 0.29 | 0.21 | 0.28 | 0.35 | 0.28 | 0.37 | 0.45 |
| 0.6 | 0.13 | 0.18 | 0.23 | 0.15 | 0.21 | 0.27 | 0.19 | 0.26 | 0.33 | 0.27 | 0.35 | 0.43 |
| 0.5 | 0.11 | 0.16 | 0.21 | 0.13 | 0.19 | 0.24 | 0.17 | 0.23 | 0.30 | 0.24 | 0.32 | 0.40 |
| 0.4 | 0.10 | 0.14 | 0.18 | 0.12 | 0.16 | 0.21 | 0.15 | 0.21 | 0.27 | 0.22 | 0.29 | 0.37 |
| 0.3 | 0.08 | 0.11 | 0.15 | 0.10 | 0.14 | 0.18 | 0.13 | 0.18 | 0.23 | 0.19 | 0.26 | 0.33 |
| 0.2 | 0.06 | 0.08 | 0.11 | 0.07 | 0.10 | 0.13 | 0.10 | 0.14 | 0.18 | 0.15 | 0.21 | 0.27 |
| 0.1 | 0.03 | 0.05 | 0.06 | 0.04 | 0.06 | 0.08 | 0.06 | 0.08 | 0.11 | 0.10 | 0.14 | 0.18 |

| 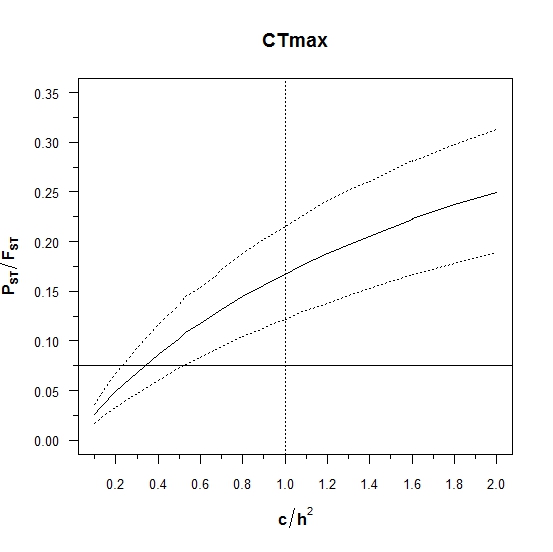 | 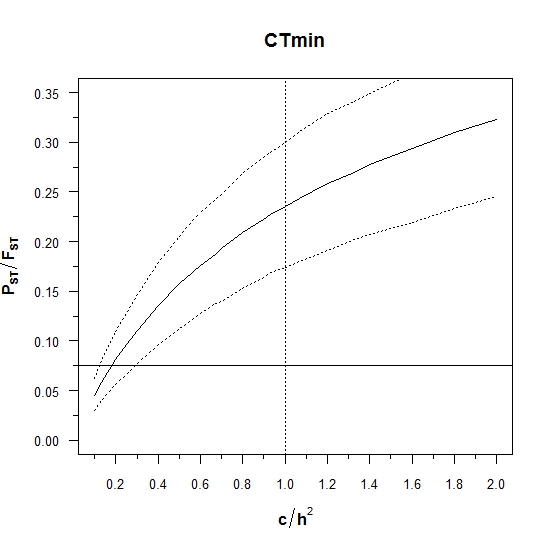 |
| --- | --- |

**Figure S1.** Comparison between neutral differentiation and the estimate of P_ST_ depends on the c/h^2^ ratio. CT_max_ (left), CT_min_ (right). In each plot the dashed vertical line denotes the ‘null assumption’ c=h^2^ for estimating P_ST._ The horizontal solid line marks the upper confidence estimate of the neutral divergence estimated as F_ST_ (= 0.075). Estimates of P_ST_ and its lower and upper 95% confidence intervals are plotted. For both CT_max_ and CT_min_, P_ST_ clearly differed from F_ST_ under the null hypothesis (P_ST_ > F_ST_).
